# Suppressing *Cis*/*Trans* ‘Ring‐Flipping’ in Organoaluminium(III)‐2‐Pyridyl Dimers–Design Strategies Towards Lewis Acid Catalysts for Alkene Oligomerisation

**DOI:** 10.1002/chem.202303872

**Published:** 2024-04-04

**Authors:** Dipanjana Choudhury, Ching Ching Lam, Nadia L. Farag, Jonathan Slaughter, Andrew D. Bond, Jonathan M. Goodman, Dominic S. Wright

**Affiliations:** ^1^ Yusuf Hamied Department of Chemistry University of Cambridge Lensfield Road Cambridge CB2 1EW; ^2^ The Faraday Institution Quad One Harwell Science and Innovation Campus Didcot OX11 0RA United Kingdom

**Keywords:** organoaluminum(III), dimerization, isomerism, cations, alkene activation

## Abstract

Owing to its high natural abundance compared to the commonly used transition (precious) metals, as well as its high Lewis acidity and ability to change oxidation state, aluminium has recently been explored as the basis for a range of single‐site catalysts. This paper aims to establish the ground rules for the development of a new type of cationic alkene oligomerisation catalyst containing two Al(III) ions, with the potential to act co‐operatively in stereoselective assembly. Five new dimers of the type [R_2_Al(2‐py′)]_2_ (R=Me, ^*i*^Bu; py′=substituted pyridyl group) with different substituents on the Al atoms and pyridyl rings have been synthesised. The formation of the undesired *cis* isomers can be suppressed by the presence of substituents on the 6‐position of the pyridyl ring due to steric congestion, with DFT calculations showing that the selection of the *trans* isomer is thermodynamically controlled. Calculations show that demethylation of the dimers [Me_2_Al(2‐py′)]_2_ with Ph_3_C^+^ to the cations [{MeAl(2‐py’)}_2_(μ‐Me)]^+^ is highly favourable and that the desired *trans* disposition of the 2‐pyridyl ring units is influenced by steric effects. Preliminary experimental studies confirm that demethylation of [Me_2_Al(6‐MeO‐2‐py)]_2_ can be achieved using [Ph_3_C][B(C_6_F_5_)_4_].

## Graphical abstract

**Controlling the** ***cis*** **/*trans* isomerism** of the dimers [R_2_Al(μ‐2‐py’)]_2_ is the key to the development of co‐operative cationic Al(III) catalysts that may offer stereo‐control of alkene insertion during polymerization, in a similar way to established Zr(IV)‐based systems. Substitution at the 6‐position of the 2‐py’ groups favors the desired *C*_2_‐symmetric *trans* isomer and the stability of the cations [{RAl(2‐py’)}_2_(μ‐Me)]^+^ formed by demethylation.

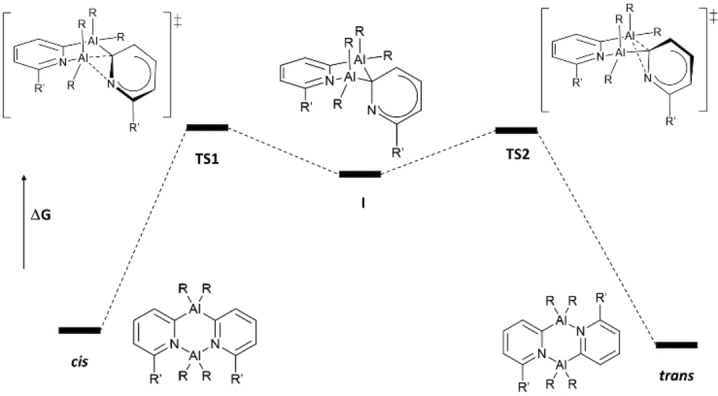

## Introduction

Transition metals have maintained a central role in the fields of synthesis and catalysis for decades. Owing to their easily accessible and highly variable oxidation states, transition metals have been considered to possess richer chemistry than that of main group metals.^[1]^ However, precious (4d and 5d) transition metals have low natural abundance and are expensive, and their extraction is often not environmentally benign. The increasing drive towards sustainability has prompted researchers to explore the reactivity of complexes of relatively cheaper main group metals. Until Power's seminal 2010 review *“Main‐group elements as transition metals”*,^[1]^ the reactivity of the heavier main group elements was largely thought to resemble that of their lighter congeners.^[2]^ However, it was recognised that despite the lack of accessible d‐orbitals, unoccupied frontier orbitals in main group atoms can participate in back‐bonding and can therefore be used in small molecule activation.^[1]^

The area of main group *redox catalysis* (involving reversible changes in oxidation state) is still very much at its early stages, largely because fully reversible systems based on main group elements are difficult to stabilise, especially bearing in mind the reduction in bond energies on descending a p‐block group (cf. the increase in bond energy for a transition metal triad). However, examples of *static catalysis* (in which there is no change in oxidation state) have been established for some time.

As the most abundant metal in the Earh crust and with the potential to cycle between the I‐ and III‐oxidation states, aluminium is an attractive candidate for catalytic systems. The first example of static catalysis involving Al(III) was introduced almost 100 years ago in the Meerwein–Ponndorf–Verley process in which ketones and aldehydes are reduced to the corresponding alcohols using Al(O^i^Pr)_3_.^[3]^ Static catalytic dehydrocoupling reactions using Group 13 III‐oxidation state reagents have also been found to function in a mechanistically similar way to transition metal catalysts.^[4]^ Over the past few years, there has been a significant focus on molecular activation using Al(I), with the aim of obtaining redox catalysis involving the Al(I)/Al(III) couple.[^8^, ^9^] So far, however, no catalytic examples have been reported.

Most relevant to the current work is small‐molecule activation using cationic Al(III) species. A study by Harder *et al*. has shown that the Al(III) cation [{(DippNCMe)_2_CH}Al^III^Me]^+^ (Dipp=2,6‐diisopropylphenyl) can reversibly bind alkenes at the cationic Al(III) atom.^[10]^ An important potential application of this work is in alkene oligomerisation/polymerization in a similar manner to well established single‐site Zr(IV)‐based Ziegler‐Natta catalysts. Also pertinent to the current study, we have previously explored an extensive range of main group tris‐pyridyl complexes and used these as ligands with various main group and transition metal elements.[^5^, ^6^] This previous work has shown that main group ligands of this type are not only robust enough to develop new coordination chemistry, but their metal complexes are also stable enough to be used in catalysis.^[7]^

**Scheme 1:**
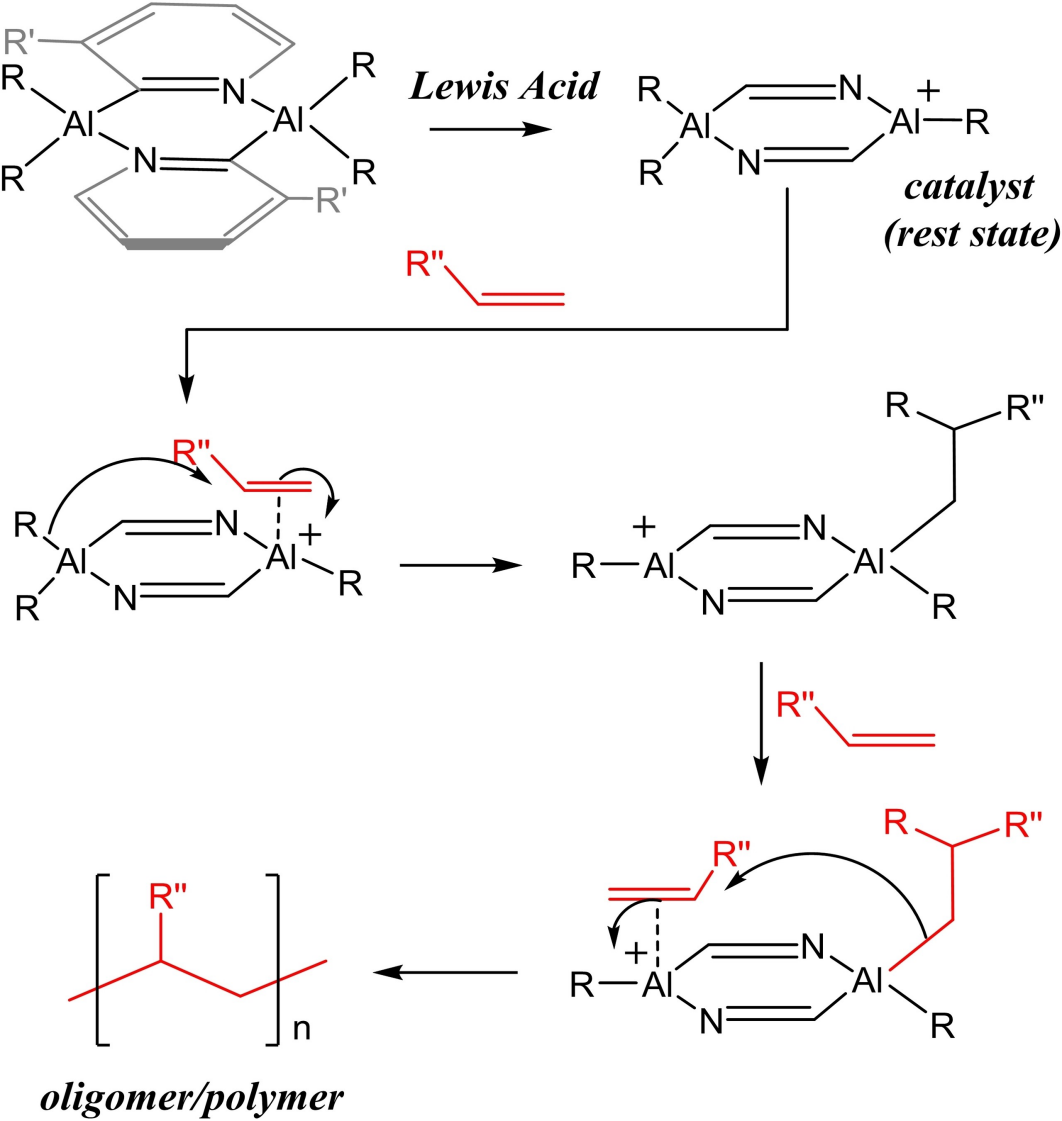
Potential oligomerisation/polymerization mechanism using organoaluminium dimeric cationic species.

In the current work, we explore the synthesis and reactivity of organoaluminium dimers [R_2_Al(2‐py′)]_2_ (where R is an alkyl group and py′ is an unsubstituted or substituted pyridyl group)^[14]^ as a potential framework to support new cationic Al(III) species. The presence of two coordination sites in cations derived from de‐alkylation provides the potential for both Al(III) ion to function cooperatively in alkene polymerisation (Scheme 1). One promise of this approach is that the installation of substituents in the 2‐py’ groups might provide stereoselectivity in the alkene insertion reactions at either Al(III) atom (allowing syndiotactic or isotactic chain growth).

## Results and Discussion

### Synthetic and Structural Studies

**Scheme 2:**
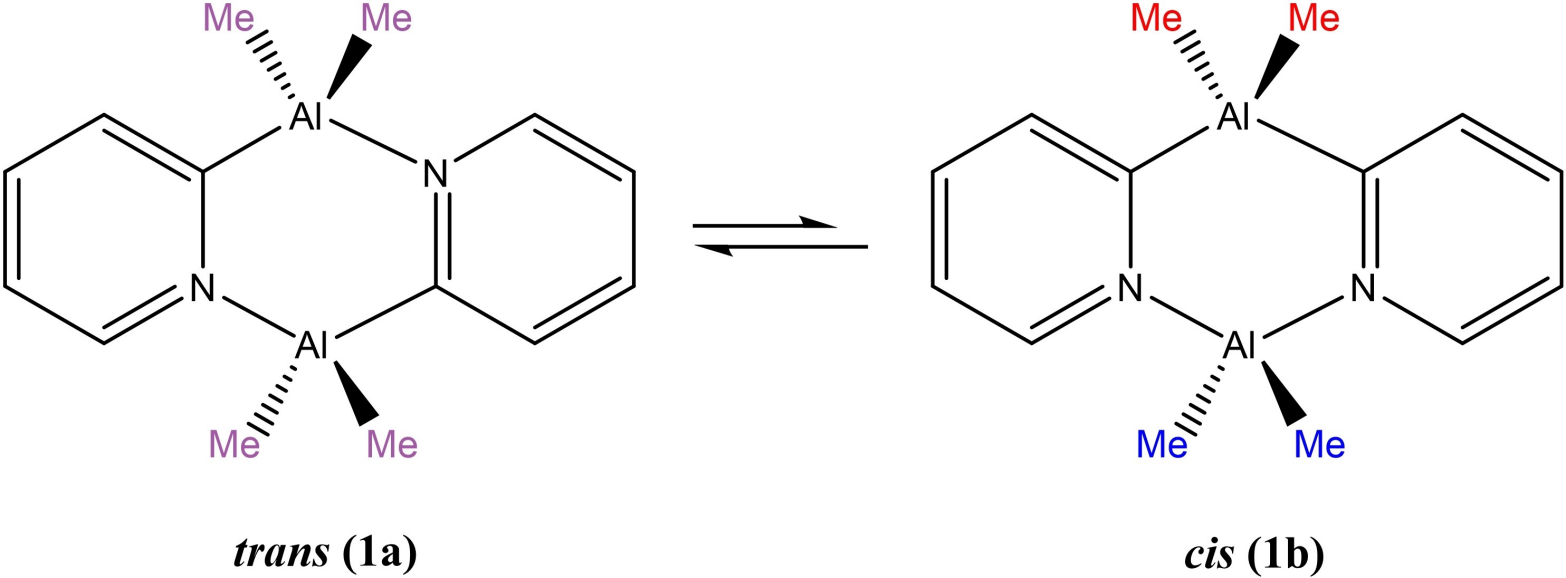
(a) Equilibrium between the *trans* (**1 a**) and *cis* (**1 b**) isomers of [Me_2_Al(2‐py)]_2_ (**1**). The presence of one resonance for the Me‐groups in the *trans* isomer and two resonances for the *cis* is indicated by the colouring.

In a previous communication we showed that the significant problem in establishing dimers of the type [R_2_Al(2‐py′)]_2_ as scaffolds for Al(III)‐based alkene activation is the presence of *cis* and *trans* isomers in solution.^[8]^ In the case of [Me_2_Al(2‐py)]_2_ (**1**), NMR studies indicate that the thermodynamically more stable *trans* isomer (**1 a**) dominates, with the *trans to cis* isomer (**1 b**) ratio being 85 : 15 (Scheme 2). This is also seen in the solid‐state structure of **1** as mixed site occupancy of the Al‐bonded C‐ and N‐atoms within the (AlCN)_2_ ring unit. Clearly, in order to preserve the *C*_2_‐symmetry necessary for stereoselective alkene insertion during polymerisation, formation of the *cis* isomer needs to be suppressed. An important NMR handle in this regard is the presence of one resonance for the Al(III) bonded alkyl groups in the *trans* isomer and two resonances for the *cis*, allowing rapid assessment of the isomeric composition.

**Scheme 3:**
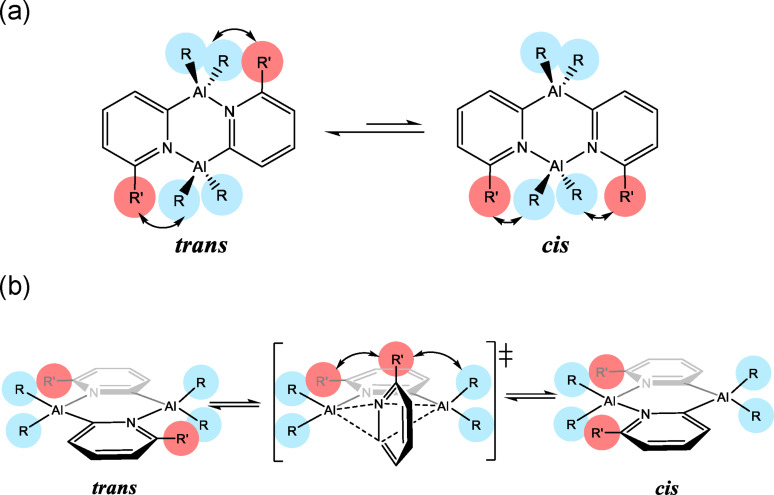
(a) Steric effect of substitution at the 6‐position (R’) in the *cis* and *trans* isomers, and (b) the steric effect of the Al‐bonded groups (R) on the (assumed) concerted transition state for ‘ring flipping’.

Our initial studies in the current work therefore set out to explore how substitution of the pyridyl ring units might affect this *cis*–*trans* equilibrium, with a goal to favour the *trans* isomer. We reasoned that the substitution of the 2‐pyridyl rings at the 6‐position is likely to influence the molecular conformation, and therefore the relative thermodynamic stabilities of the *cis* and *trans* isomers ‐ since the steric interactions with the Al‐bonded alkyl groups will be exacerbated in the *cis* isomer in which the most sterically crowded Al(III) atom is N,N‐bonded (Scheme 3a). In addition, increasing the steric bulk of the Al‐bonded alkyl groups could potentially have a significant effect on the activation energy for the (initially assumed) intramolecular ‘ring flipping’ process (Scheme 3b).^[15]^

**Scheme 4:**
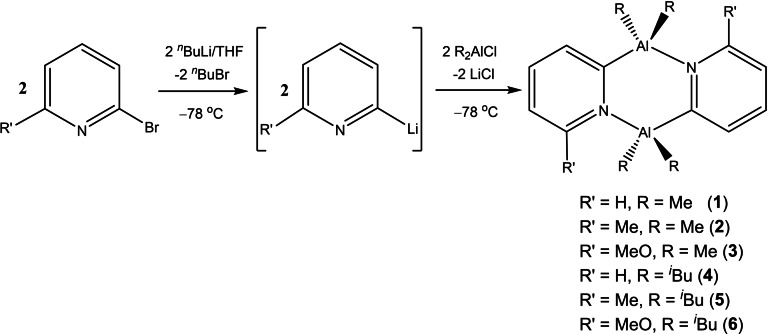
Synthetic route used to obtain the dimers [R_2_Al(2‐py′)]_2_, showing the numbering scheme used in the text.

To explore the effects of changing the steric demands of the Al‐bonded (R) and 6‐substituents of the pyridyl groups (R’), a series of new dimers was prepared using a similar procedure to that employed previously for **1** (Scheme 4).^[8]^ The new dimers (**2**–**6**) were characterised using multinuclear solution‐state NMR spectroscopy (^1^H, ^13^C, ^27^Al) prior to obtaining their solid‐state structures using single‐crystal X‐ray diffraction. It should be noted that the structure determinations of **2** and **6** are of very limited quality and have some problems with disorder and limited diffraction to higher angles. Nonetheless, their overall arrangements are unambiguous.

**Figure 1:**
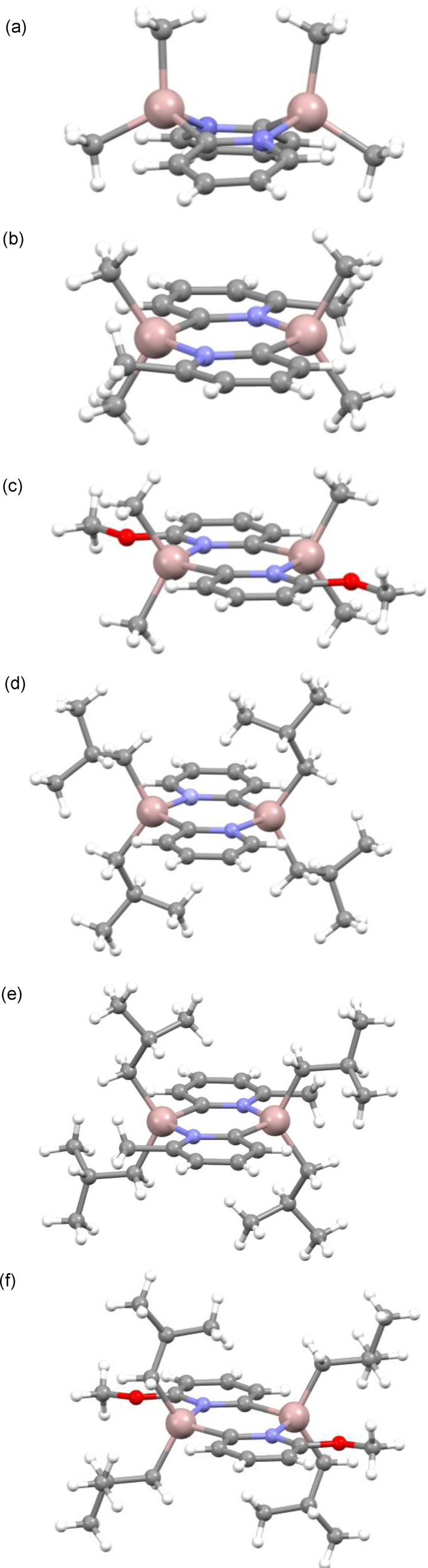
Solid‐state structures of (a) [Me_2_Al(2‐py)]_2_ (**1**), (b) [Me_2_Al(6‐Me‐2‐py)]_2_ (**2**), (c) [Me_2_Al(6‐MeO‐2‐py)]_2_ (**3**), (d) [^*i*^Bu_2_Al(2‐py)]_2_ (**4**), (e) [^*i*^Bu_2_Al(6‐Me‐2‐py)]_2_ (**5**) and (f) [^*i*^Bu_2_Al(6‐MeO‐2‐py)]_2_ (**6**). The structure of **1** has been reported previously and is included here for comparison (CCDC ref. code REWPEP). Selected bond lengths and angles can be found in the ESI (Table S2). In **1** and **4**, site disorder of the C and N atoms within the (AlCN)_2_ rings is omitted for clarity.

The impact of the greater steric demands of the R and R’ ligands can be seen directly from the solid‐state structures of **1**–**6** (Figure 1). Whereas the previously reported dimer **1** has a boat‐shaped Al_2_(C^…^N)_2_ ring, with a puckering angle of *ca*. 140°, the new dimers in **2**–**6** all have planar ring units.^[8]^ This change in conformation is consistent with the minimisation of repulsion between the R and R’ groups and/or transannular repulsion between the R groups. Despite the differences in the substituents, there are only comparatively small variations in the metric parameters within **2**–**6**. In the 6‐MeO substituted dimers **3** and **6** there are no obvious indications of significant Al•••O(Me) interactions [the Al•••O range 2.789(3)–2.808(5) Å being well outside the values expected for donor–acceptor bonds]. From the perspective of X‐ray diffraction, the presence of the substituent in **2**, **3**, **5** and **6** clearly establishes the *trans* orientation of the pyridyl rings. In **1** and **4**, however, where there is no substituent, the distinction between N and C atoms in the pyridyl rings is unclear, and the N/C atoms are modelled as disordered. In practice, this prevents any reliable conclusions concerning the potential presence of *cis* or *trans* isomers in these two crystal structures.

**Figure 2:**
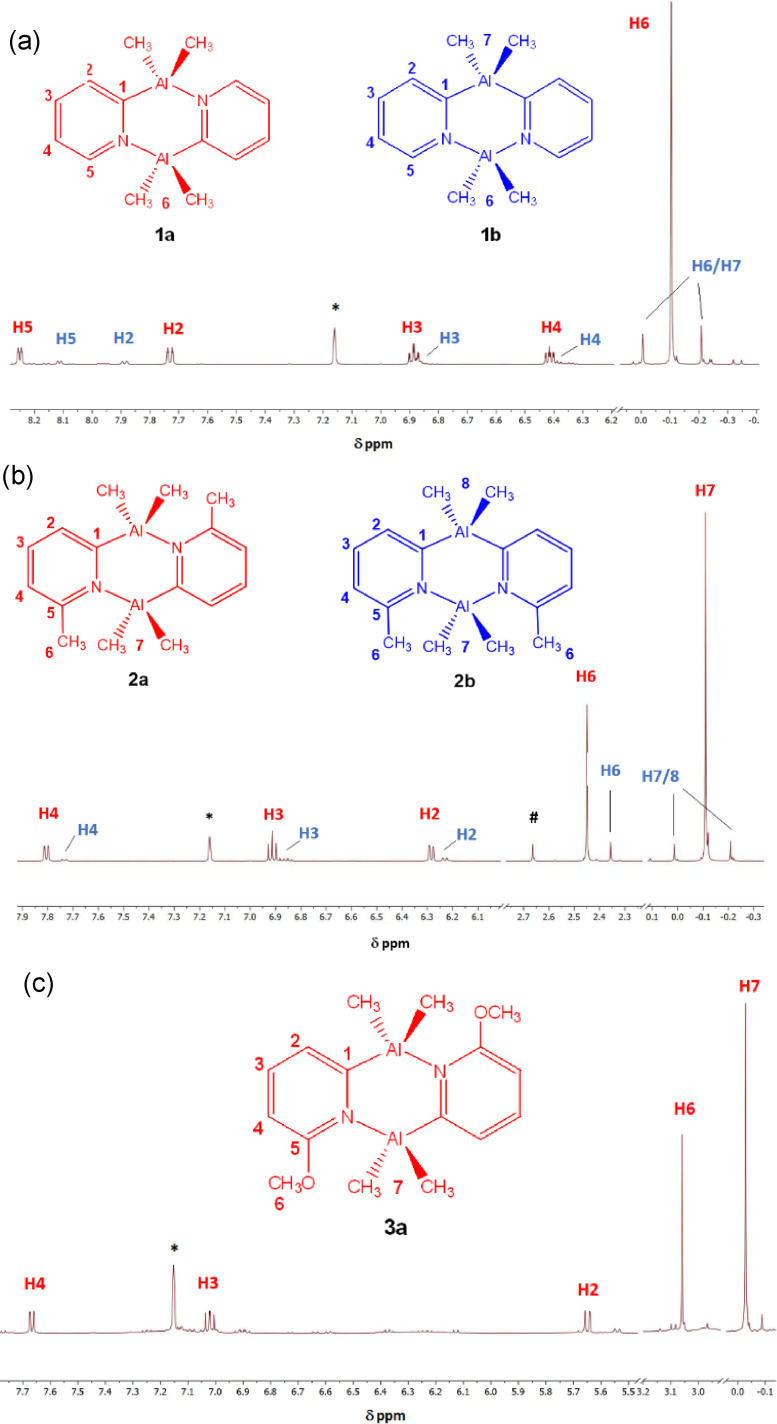
^1^H NMR spectra (25 °C, d_6_‐benzene, 500.1 MHz) of pre‐isolated (a) **1**, (b) **2** and (c) **3**. (*d_6_‐benzene. # in compound **2** is a persistent impurity which could not be assigned).

The room‐temperature ^1^H NMR spectra of isolated **1**–**3** in d_6_‐benzene are shown in Figure 2. In line with our expectations, integration of the well‐separated Al‐bonded Me‐resonances shows an observable reduction from 15 %(*cis*):85 %(*trans*) in **1** to 8 %(*cis*):92 %(*trans*) in **2**, while in **3** there is little or no sign of the *cis* isomer. Thus, at first sight it does appear that the introduction of groups at the 6‐position of the 2‐pyridyl ligands supresses the *cis* isomer in solution.

**Figure 3:**
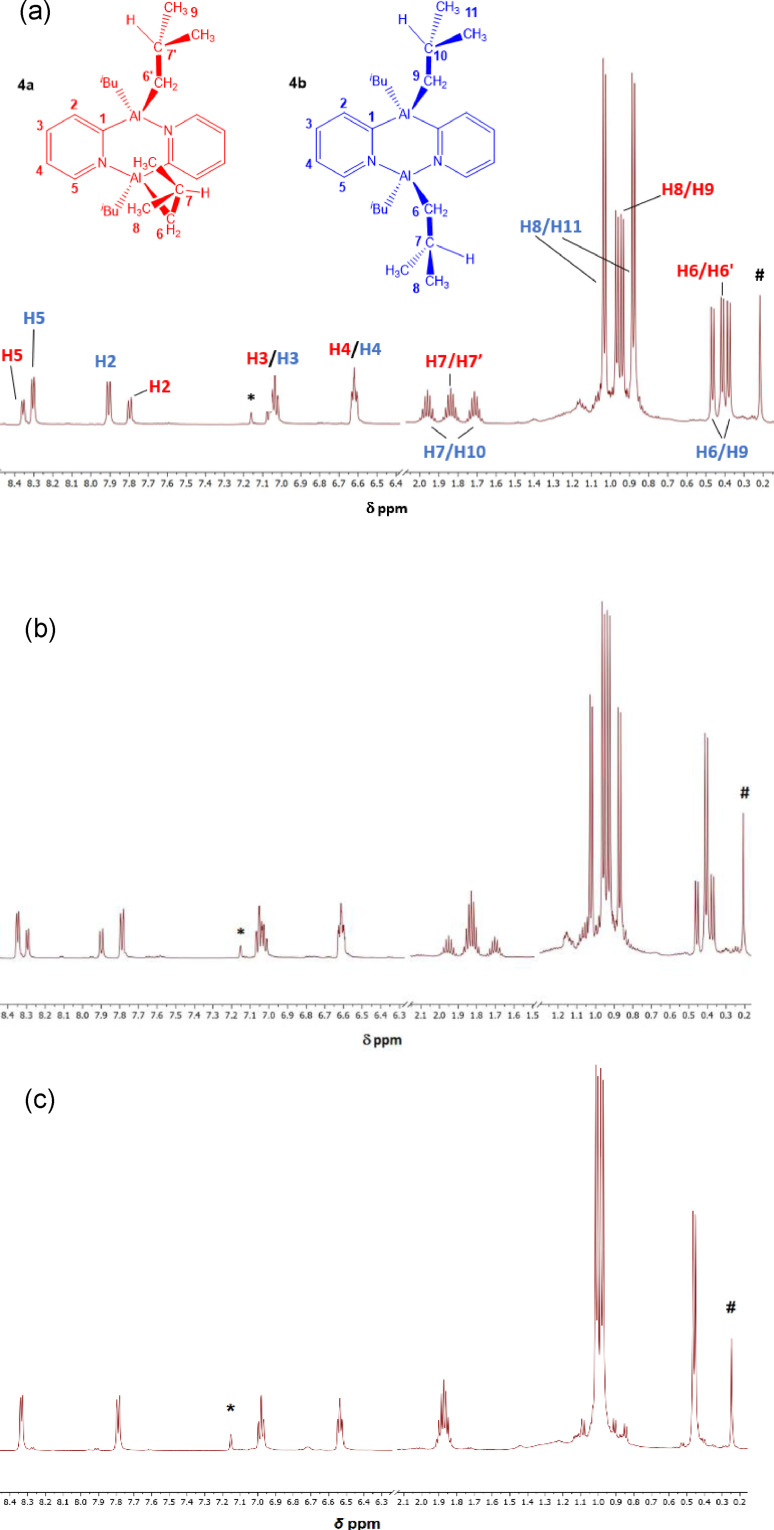
^1^H NMR spectra (25 °C, d_6_‐benzene, 500.1 MHz) of (a) freshly isolated **4** at 25 °C, (b) after 11 days at room temperature and (c) after heating the sample in (b) to 60 °C for 48 h. *d_6_‐benzene, # vacuum grease.

The room‐temperature ^1^H NMR spectra of isolated **5** and **6** in d_6_‐benzene show a similar trend to that seen for **1**–**3**, now with little or no *cis* isomer being detected in each (see ESI, Figures S9 and S12). Some of the most interesting behaviour in solution was observed for dimer **4**. The ^1^H spectrum of a freshly isolated sample shows the presence of 70 % *cis* and 30 % *trans* isomer (Figure 3a). After storage at room‐temperature for 11 days, however, there is a significant decrease in the amount of the *cis* isomer (now *ca*. 50 %:50 %) (Figure 3b). Heating this sample to 60 °C for 48 h leads to the almost exclusive formation of the *trans* isomer (Figure 3c). These data are in accord with expectations that there is likely to be a kinetic as well as a thermodynamic basis to the selection of either isomer. Clearly, in the case of **4** there is a relatively large activation energy for conversion of the *cis* to the *trans* isomer. A further feature seen in the room‐temperature ^1^H NMR spectrum of **4** as well as those of **5** and **6** is the presence of two Me‐environments in the ^*i*^Bu groups for the *trans* isomer [H8(d.)/H9(d.)]. This is due to the disposition of the ^*i*^Bu groups *exo* and *endo* with respect to the (AlCN)_2_ ring units and shows that the structure of this isomer is essentially the same as that in the solid‐state structures (see Figure 1d, e and f). In contrast, since only two ^*i*^Bu Me‐environments are found for the *cis* isomer of **4** [H8(d.)/H11(d.)] these groups are either spatially equivalent or the activation energy for *exo*/*endo* inversion is low. The fact that no coalescence of the Me‐resonances of the *trans* isomer occurs even at 100 °C in d_8_‐toluene suggests that the former is more likely to be the case.

### Theoretical Studies of the Ring‐Flipping Mechanism

DFT calculations on the dimers **1**, **2**, **4** and **5** were performed to assess the influence of the R and R’ groups on the kinetics and thermodynamics at the ωB97X‐D/6‐311++G(d,p)//B3LYP‐D3/6‐31G(d) level of theory. (See ESI Computational Study section for the full details).[^9^, ^10^, ^11^, ^12^, ^13^, ^14^] Dimer **1** was studied in detail to pinpoint the probable mechanism of the ring‐flipping process. Three possible mechanisms were explored, *(i)* a dissociative pathway involving the formation of monomers from the *trans* isomer which recombine into the *cis*; *(ii)* a concerted mechanism which was assumed to be the most likely mechanism at the beginning of the study (like that depicted in Scheme 3); *(iii)* a stepwise mechanism involving Al–N bond cleavage.

**Figure 4:**
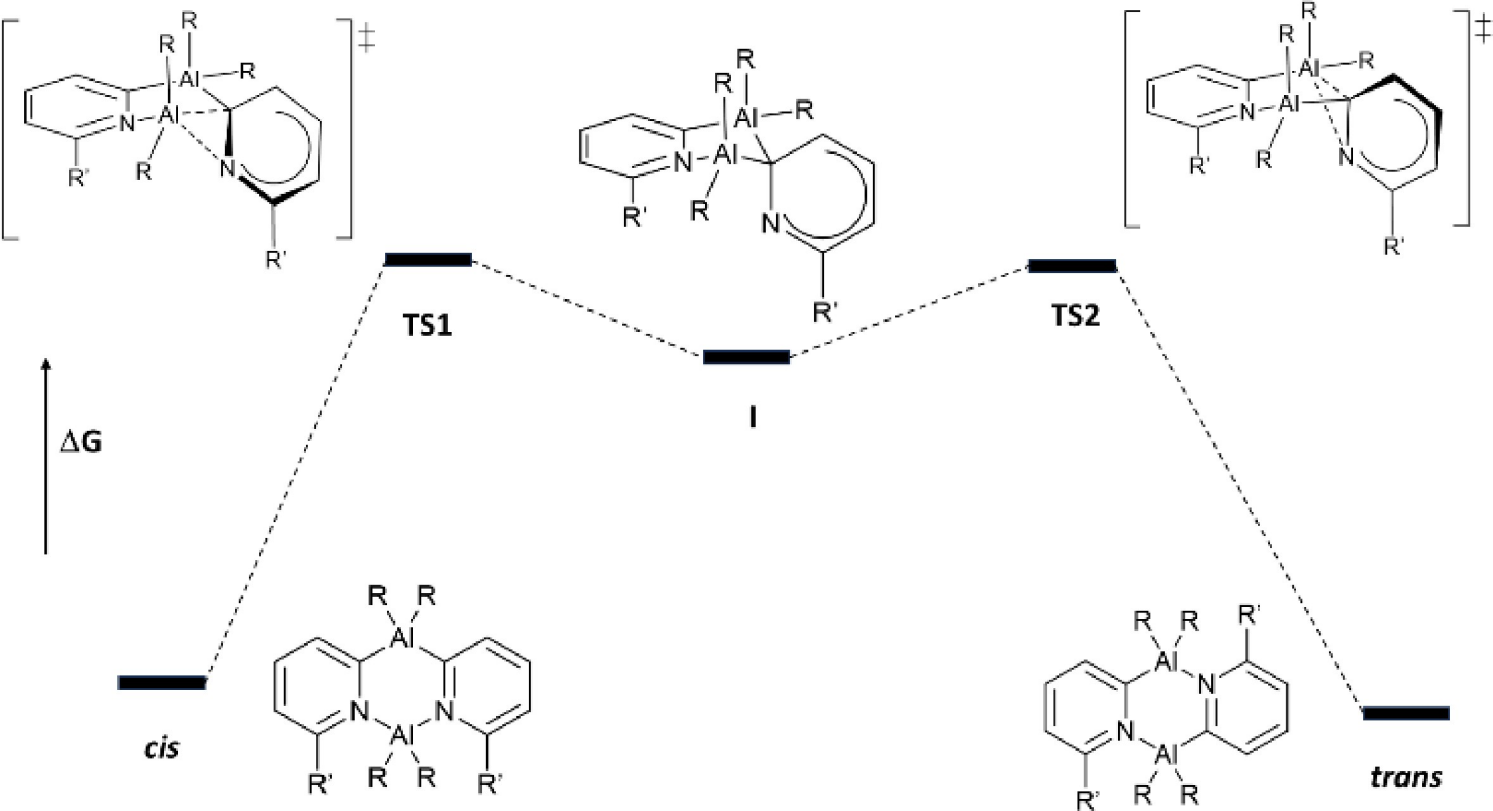
Generalised outline of the lowest‐energy two‐step mechanism of ring flipping pinpointed by DFT calculations. Further details of the geometries of the transition states and intermediates for **1**, **2**, **4** and **5** can be found in the ESI.

Contrary to our initial expectations, the calculations on **1** show that a stepwise ‘swing’ mechanism is the most kinetically favourable, as depicted in Figure 4. The first step involves cleavage of one of the Al−N bonds of the *cis* isomer, with rotation of the 2‐pyridyl group in an *anti*‐fashion (i. e., with the N‐atom downwards with respect to the initial Al_2_N_2_ ring unit) to form an intermediate (I) containing two four‐coordinate Al atoms, which then forms the new Al−N bond of the *trans* isomer in the second step. A closely related *syn*‐swing mechanism in which the N‐atom is rotated upwards with respect to the initial Al_2_N_2_ ring unit was found to be marginally less kinetically favourable. In addition, concerted pathways had far higher activation energies (ΔG^≠^=44.8–48.0 kcal mol^−1^) and the dissociation of dimers into monomers is clearly highly thermodynamically unfavourable (ΔG_dissociation_=47.7 kcal mol^−1^ for the *trans* dimer).

**Figure 5:**
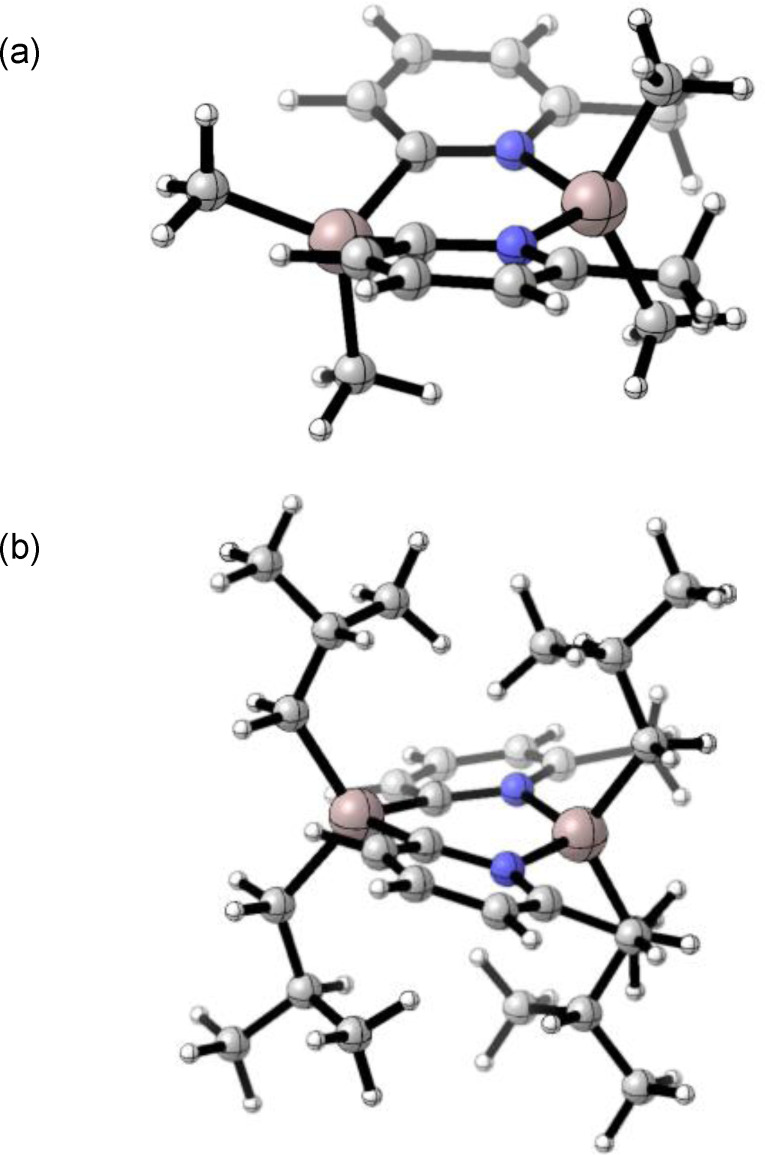
The geometry optimised structures of the *cis* isomers (a) **2 b** and (b) **4 b**, showing their puckered (AlCN)_2_ ring units. For **2 b**, the puckered ring can be converted to its inverted version via a planar transition state with an activation energy of 2.4 kcal mol^−1^ (see Figure S24).

We repeated the calculations on dimers **2**, **4** and **5**. The conformational space of dimers with ^*i*^Bu substituents has been explored thoroughly (See ESI Computational Study section for the full details).^[15]^ CONFPASS was used to guide the DFT‐reoptimisations.^[16]^ Table 1 lists the relative energies of the transition states (**TS1** and **TS2**) and intermediates (**I**) for the stepwise *anti*‐swing pathways for **1**, **2**, **4** and **5** determined by DFT calculations. Although finely balanced energetically, the data for all of the dimers show that the selection of the *trans* isomer over the *cis* is under thermodynamic control, with the *trans* isomers being the most stable and with the activation energy for *trans*→*cis* (ΔG^ǂ^_(*trans*)_=ΔG_TS1_)>*cis*→*trans* (ΔG^ǂ^_(*cis*)_=ΔG_TS1_–ΔG_*cis*_). There is a clear influence of steric factors on the thermodynamics and kinetics of the reaction. Thus, incorporation of the Me‐substituents into the 2‐pyridyl rings (**1**→**2** and **4**→**5**) results in an increase in the thermodynamic stability of the *trans* isomer over the *cis*, presumably because of the increase in steric congestion at the N‐chelated Al‐atom in the *cis* isomer. Steric effects are particularly apparent from the geometry optimised structures of the *cis* isomers **2 b** and **5 b** which possess puckered (AlCN)_2_ ring units (Figure 5), while the *trans* isomers are planar (as in their solid‐state structures). It can be noted that calculations also show that both the *cis* and *trans* isomers of **4**, containing no Me‐substituent on the 2‐pyridyl rings, also have planar (AlCN)_2_ ring units.

**Table 1:** Relative energies (kcal mol^−1^) of the transition states and intermediates involved in the lowest‐energy, two‐step ‘swing’ mechanism identified by DFT calculations. *The ΔG values are relative to the corresponding *trans* isomer, with the energy of the *trans* set to 0 kcal mol^−1^.

Dimer	ΔG_*cis*_	ΔG_TS1_	ΔG_I_	ΔG_TS2_	ΔG_*trans*_ *
**1**	2.2	27.5	23.6	25.0	0
**2**	5.4	24.7	19.8	21.7	0
**4**	2.7	29.8	25.2	26.6	0
**5**	3.3	26.6	22.4	23.8	0

The introduction of the Me‐groups into the 2‐pyridyl rings moving from **1**→**2** and **4**→**5** also results in a decrease in the energies of **TS1**, **TS2** and **I** in both cases. This is consistent qualitatively with the experimental observation of the persistence of the *cis* isomers of **1** and **4** in initially isolated samples, since their *cis* isomers have higher calculated activation energies for the conversion to *trans* (ΔG^ǂ^_(*cis*)_) than their Me‐substituted counterparts.

**Scheme 5:**
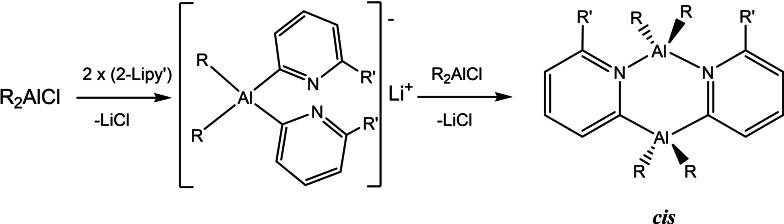
Potential reason for the initial formation of significant amounts of the *cis* isomers for **1** and **4**.

Considering the calculations alone, however, the predominant formation of the *cis* isomer in **4** in initially isolated samples is difficult to explain unless there is a kinetic origin within the reaction itself. One possibility is outlined in Scheme 5: rather than forming monomers [R_2_Al(2‐py’)] which then dimerise, formation of [R_2_Al(2‐py’)_2_]^−^ sets up the *cis* arrangement of the dimer, which then rearranges into the *trans* isomer under thermodynamic control. This pathway is likely to be most favourable for less sterically bulky 2‐py’ groups, as are present in **1** and **4** where significant amounts of the *cis* isomer are observed in the initially isolated products.

### Preliminary Studies of the Demethylation of the Dimers

**Figure 6:**
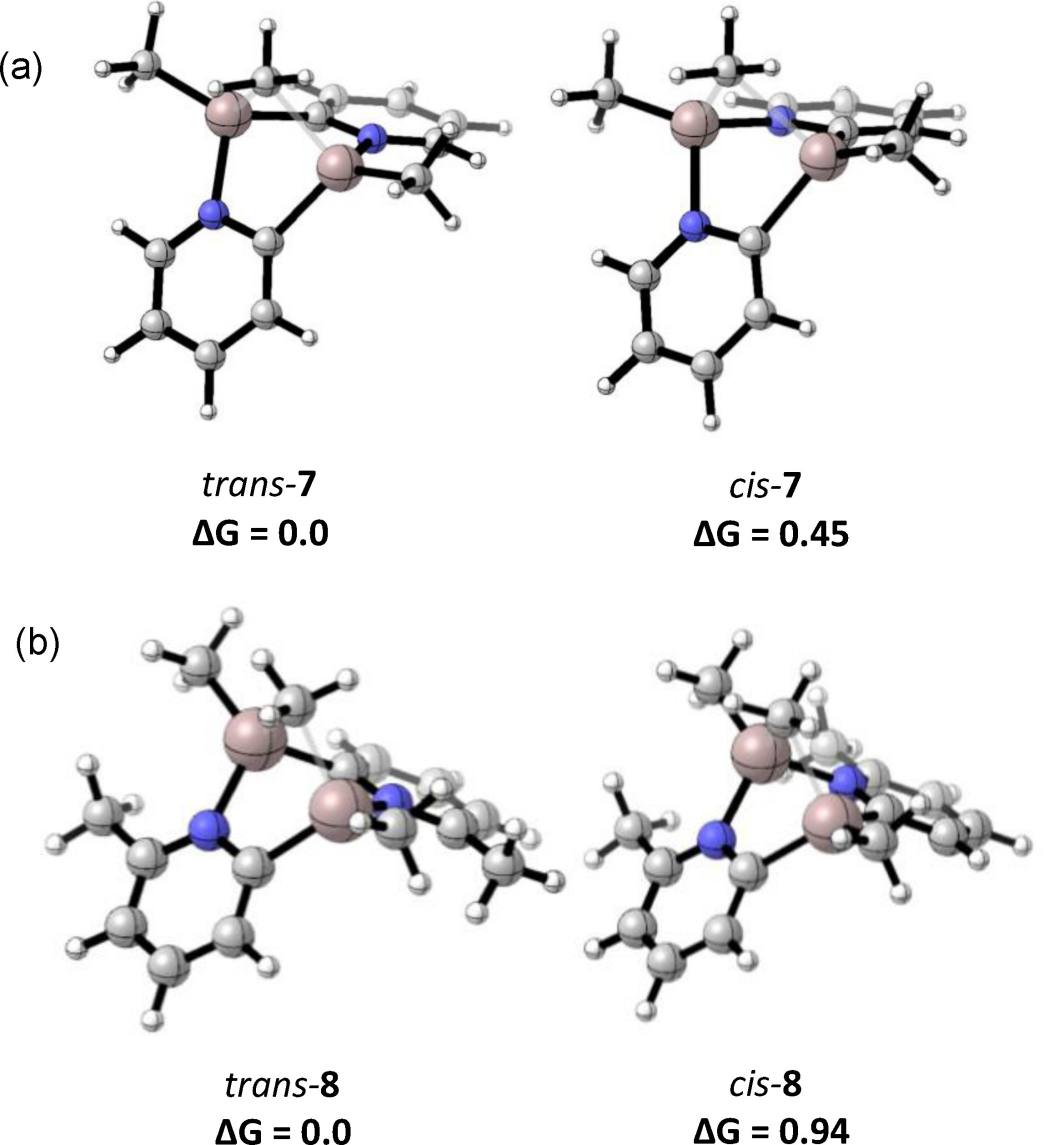
Geometry (DFT) optimised structures of the [{MeAl(2‐py’)}_2_(μ‐Me)]^+^ cations generated by single demethylation of (a) **1** and (b) **2**. ΔG values are in kcal mol^−1^.

The structures of the products of the single demethylation of dimers **1** (R=Me, R’=H) and **2** (R=R’=Me) were explored using DFT calculations at the same level as employed previously. Full details of the bond lengths, charges and bond orders can be found in the SI. Removing one of the Me‐groups from each gives the most stable cations [{MeAl(2‐py’)}_2_(μ‐Me)]^+^ (*trans*‐**7** and *trans*‐**8**, respectively) in which one of the Me‐groups moves into a bridging Al(μ‐Me)Al mode; a formal 3c–2e bond that is reminiscent of the Al(μ‐Me)Al bonding in the trimethylaluminium dimer, [Me_2_Al(μ‐Me)]_2_ (Figure 6). This arrangement is significant with respect to potential applications in alkene oligomerisation as it should stabilise the catalytic rest state (see Scheme 1), and results in nearly equal charges on the Al‐atoms. These *trans* structures are slightly more stable than the corresponding *cis* isomers (*cis*‐**7** and *cis*‐**8**), with the stability of the *trans* isomer increasing with respect to the *cis* with the introduction of the Me‐substituent on the 2‐pyridyl ring of **8** (no doubt a consequence of similar steric effects that are seen in the structures of the dimers themselves). This trend presents a further advantage in introducing substituents at the 6‐position of the 2‐pyridyl ring units, in order to retain the desired *trans* arrangement for catalysis.

**Scheme 6:**
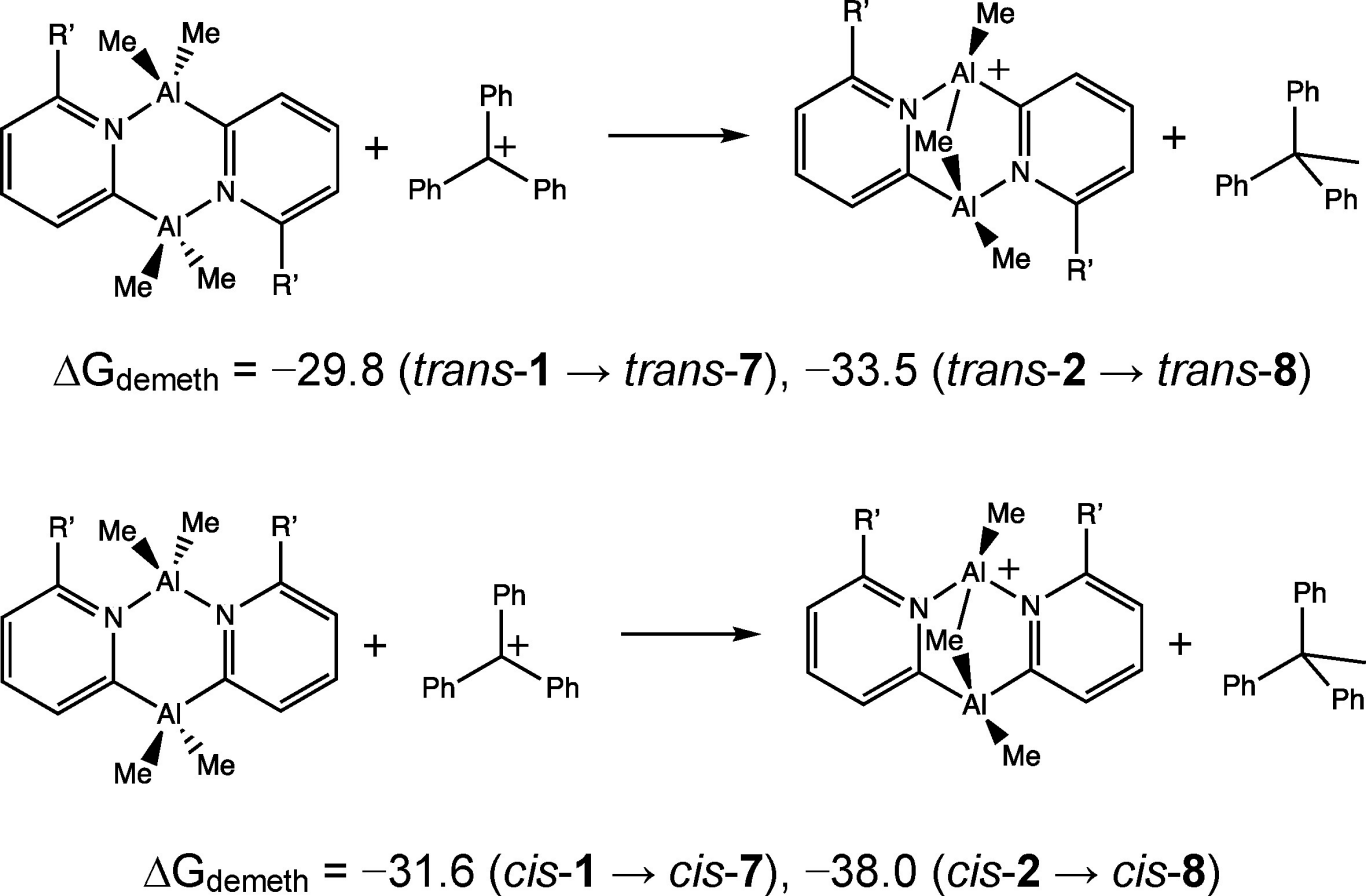
Calculated ΔG_demeth_ for demethylation of **1** and **2** to the *trans* (top) and *cis* (bottom) isomers. Values of ΔG_demeth_ are in kcal mol^−1^.

A further and potentially undesirable effect of increasing the steric crowding is seen in the calculation of ΔG_demeth_ for the demethylation of the *cis* and *trans* isomers of **1** and **2** with Ph_3_C^+^. (Scheme 6). While ΔG_demeth_ is most favourable for the *cis* isomers of **1** and **2** compared to the *trans*, ΔG_demeth_ for the *cis* isomer of **2** is significantly more favoured over the *trans* isomer compared to **1**. This is presumably due to release from steric crowding of the Me‐groups in the *cis* isomer of **2** upon demethylation. It can be noted from the previous experimental and calculational results, however, that the *trans* isomers are by far the dominant species for all of the dimers investigated (after thermal equilibration) and that the activation energies for the conversion of the *trans* isomers to the *cis* far outweigh the relatively small difference in ΔG_demeth_ between the *cis* and *trans* isomers found here (ΔG_TS2_, Table 1). Further kinetic calculations of the isomerism of *cis*‐ and *trans*‐**7** and **8** were not undertaken as part of the current study.
(1)

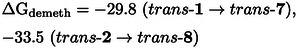



(2)

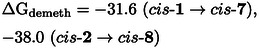




In a pilot study, the demethylation of **1** and **3** with [Ph_3_C][B(C_6_F_5_)_4_] was explored. The same Lewis acid has previously been used extensively in the formation of cationic aluminium alkyls, mainly involving dealkylation of β‐diketiminate supporting ligands.[^17^, ^18^] The synthetic procedure outlined by Jordan *et al*. was followed here.^[18]^ The yellowish brown solid product of the reaction of **3** and [Ph_3_C][B(C_6_F_5_)_4_] was isolated easily. The room‐temperature ^1^H NMR spectrum in d_6_‐benzene reveals a peak at *δ* 2.04 ppm which could be confidently attributed to Ph_3_CMe, indicating that a methyl group has been successfully extracted from the dimer **3**. The full ^1^H NMR spectrum is shown in Figure S16, in which the signals have been assigned based on the integrals. Ph_3_CMe is also observed in the *in situ* NMR spectrum of the reaction of **1** and [Ph_3_C][B(C_6_F_5_)_4_]. Efforts to obtain single crystals of the demethylation product for X‐ray analysis have thus far been unsuccessful. Once definitive evidence of the nature of the cations has been obtained their reactivity will be investigated.

## Conclusions

Building on a previous communication, this study has presented five new dimers of the type [R_2_Al(2‐py’)]_2_ containing various substituents on their Al‐atoms and at the 6‐position of the 2‐pyridyl rings. From this it has been possible to explore the effects of sterics on the relative stabilities of the *cis* and *trans* isomers. It is found that the introduction of substituents on the 2‐pyridyl rings has the greatest effect on increasing the stability of the *trans* isomers, whose *C*_2_ symmetry may form the basis for cationic Al(III)‐based catalysts for alkene polymerisation. In accord with the experimental results, DFT calculations show that the *trans* isomers are always thermodynamically preferred. An unexpected two‐step ‘swing’ mechanism is involved in the isomerisation process. Theoretical investigations show that demethylation of the dimers with Ph_3_C^+^ to give model cations of the type [{MeAl(2‐py’)}_2_(μ‐Me)]^+^ is highly thermodynamically favourable and that the influence of sterics is also important in regard to the *cis*/*trans* disposition of the 2‐pyridyl ring units of the cations. Preliminary studies of the demethylation of one of these dimers shows that demethylation does occur, although the exact nature of the species formed is not yet fully established.

In future work we will explore the use of the cations as polymerization catalysts in a similar manner to Ziegler–Natta catalysis using Zr(IV). The presence of two potential catalytic Al(III)‐sites for alkene activation provides a possible mechanism in which the cationic Al(III) ion alternates as the polymerisation proceeds (highlighted Scheme 1), which could permit stereo‐control over the insertion of the alkene units.

## Supporting Information

The authors have cited additional references within the Supporting Information. Deposition Number(s) (https://www.ccdc.cam.ac.uk/services/structures doi:10.1002/chem.202303872) 2290737 (for **2**), 2290739 (for **3**), 2290741 (for **4**), 2290740 (for **5**), 2290738 (for **6**) contain the supplementary crystallographic data for this paper. These data are provided free of charge by the joint Cambridge Crystallographic Data Centre and Fachinformationszentrum Karlsruhe (http://www.ccdc.cam.ac.uk/structures).

## Conflict of interests

The authors declare no conflict of interest.

1

## Supporting information

As a service to our authors and readers, this journal provides supporting information supplied by the authors. Such materials are peer reviewed and may be re‐organized for online delivery, but are not copy‐edited or typeset. Technical support issues arising from supporting information (other than missing files) should be addressed to the authors.

Supporting Information

## Data Availability

The data that support the findings of this study are available in the supplementary material of this article.
