# Supplementary material for: Suppressing Cis/Trans ‘Ring‐Flipping’ in Organoaluminium(III)‐2‐Pyridyl Dimers–Design Strategies Towards Lewis Acid Catalysts for Alkene Oligomerisation
Source: Chemistry. 2024 Apr 4;30(28):e202303872. doi: 10.1002/chem.202303872 (PMC11497236; doi:10.1002/chem.202303872)
Supplement: Supplementary file 1 — Supporting Information [file CHEM-30-e202303872-s001.pdf]

Chemistry–A European Journal

Supporting Information

Suppressing *Cis/Trans* 'Ring-Flipping' in Organoaluminium(III)-2-Pyridyl Dimers–Design Strategies Towards Lewis Acid Catalysts for Alkene Oligomerisation

Dipanjana Choudhury, Ching Ching Lam, Nadia L. Farag, Jonathan Slaughter, Andrew D. Bond, Jonathan M. Goodman,* and Dominic S. Wright*

Supporting Information

Table of Contents

Experimental Section 2
Synthesis of Dimers 2
Spectroscopy 5
X-ray Crystallography Data 21
Structure refinement details 21
Computational study 25
Computational methods 2
Mechanistic study 25
Key structures 26
Computational Data 33

Experimental Section

All synthetic processes were undertaken using standard Schlenk techniques under a dry nitrogen atmosphere using oven-dried glassware. Starting materials were acquired from commercial suppliers (Aldrich, Acros Organics, Alfa Aesar, Manchester Organics and Fluorochem) and used as received, unless otherwise specified. Toluene and *n*-hexanes were dried using sodium and distilled under nitrogen and THF was distilled over Na/benzophenone. Lower temperatures in synthesis were attained using a dry ice/acetone (−78 ℃) bath. All air-sensitive materials were handled in a nitrogen-filled glovebox (Saffron type α). ^{1}H, ^{13}C, ^{27}Al, ^{11}B, and ^{19}F NMR spectra were obtained on a Bruker Avance III HD 500 MHz Smart Probe spectrometer. For ^{1}H and ^{13}C NMR, chemical shifts are internally referenced to the deuterated solvent and calculated relative to TMS. The internal reference standard used for ^{27}Al NMR is $AlCl_3.6H_2O$, for ^{11}B NMR it is $BF_3.OEt_2$ in $CDCl_3$, and for ^{19}F NMR it is $CFCl_3$. Single-Crystal X-ray diffraction data were obtained using either a Bruker D8-QUEST PHOTON-100 diffractometer (Cu radiation) or a Nonius KappaCCD diffractometer (Mo radiation).

Computational methods

Density Functional Theory (DFT) calculations were carried out using Gaussian 16 (Revision A.03).[6] Structural optimisations were performed at the B3LYP-D3/6-31G(d) level of theory.[7–10] Frequency analyses were conducted to validate the optimised structures, ensuring they corresponded to either energy minima or first-order saddle points on the potential surface. Single-point energy values of optimised structures were computed at the ωB97X-D/6-311++G(d,p) level of theory.[11]

The conformational space has been studied thoroughly when for dimers with ^{i}Bu substituents. We used the crystal structure of the *trans* R=iBu/R'=H complex as the starting point and replaced Al with Si and N with C to produce a pseudo structure for conformational searching. Conformational searches were conducted in MacroModel (v11.7) with Maestro (release 2021-04)[12] using the OPLS4 force field[13] with the mixed torsional / low-mode sampling method. A setting of 1000 as the maximum number of steps and the maximum number of steps per rotatable bond was used. Conformers within an energy window of 5.0 kcal mol^{-1} were saved for further analyses. First 20.5% of the conformational searching output file at the DFT level (ωB97X-D/6-311++G(d,p)//B3LYP-D3/6-31G(d)) following the priority list from CONFPASS.[14] A %Conf of 81.4% was achieved, implying that the re-optimisation process can be terminated with confidence that the global minimum has been obtained.

Synthesis of Dimers

Synthesis of [Me$_2$Al(2-py)]$_2$ (**1**): was carried out according to the previously published literature procedure.[1] Data obtained in the current study. Yield 0.330 g, 24%.
^{1}H NMR (500.1 MHz, 25 ℃, d$_6$-benzene): *trans* isomer (85%) *δ* 8.25 (dt, 2H, H(5)), 7.73 (dt, 2H, H(3)), 6.88 (td, 2H, H(4)), 6.41 (mult, 2H, H(2), −0.10 (s, 12H, Me$_2$Al). *cis* isomer (15%) *δ* 8.11 (dt, 2H, H(5)), 7.89 (dt, 2H, H(3)), 6.89 (td, 2H, H(4)), 6.39 (mult, 2H, H(2)), 0.01 (s, 6H, Me$_2$Al), −0.21 (s, 6H, Me$_2$Al).
^{13}C NMR (126 MHz, 25 ℃, d$_6$-benzene): *trans* isomer *δ* 147.0 (C(1)), 136.6 (C(5)), 135.6 (C(3)), 122.8 (C(4)), 122.8 (C(2)), −7.8 (bd, C(6)). *cis* isomer *δ* 146.15 (C(1)), 136.5 (C(5)), 136.4 (C(3)), 122.2 (C(4)), 122.2 (C(2)), −7.8 (bd, C(6) & C(7)).
^{27}Al NMR (130.3 MHz, 25 ℃, d$_6$-benzene): *δ* 158.2.

Synthesis of [Me$_2$Al(6-Me-2-py)]$_2$ (**2**): 2-Bromo-6-methylpyridine (1.14 mL, 10 mmol) was dissolved in 20 mL of THF. The resultant solution was cooled to −78 ℃ and *n*-BuLi (1.6 M in hexanes, 6.25 mL, 10 mmol) was added dropwise; the reaction was stirred for 3 h. Subsequently, dimethylaluminium chloride (1 M in hexanes, 10 mL, 10 mmol) was added dropwise at −78 ℃. The reaction was allowed to warm overnight under nitrogen atmosphere. The solvent from the resulting brown solution was removed *in vacuo* and replaced with 40 mL of toluene. The mixture was then filtered, and the filtrate was concentrated *in vacuo* until a precipitate was formed. The product was re-dissolved with gentle heating and refrigerated at 5 ℃ overnight to yield a colourless crystalline product. The solvent was removed via cannula and the product was washed with n-pentane (5 mL) and dried *in vacuo*. Yield 0.155 g, 12%.
^{1}H NMR (500 MHz, 25 ℃, d$_6$-benzene): *trans* isomer (85%) *δ* 7.80 (dd, 2H, H(3)), 6.91 (t, 2H, H(4)), 6.28 (dd, 2H, H(2)), 2.45 (s, 6H, H(6)), −0.11 (s, 12H, Me$_2$Al). *cis* isomer (15%) *δ* 7.73 (dd, 2H, H(3)), 6.87 (t, 2H, H(4)), 6.23 (dd, 2H, H(2)), 2.66 (s, 3H, H(6)), 2.35 (s, 3H, H(6)), 0.01 (s, 6H, H(7) & H(8)), −0.21 (s, 6H, H(7) & H(8)).
^{13}C NMR (126 MHz, 25 ℃, d$_6$-benzene): *trans* isomer *δ* 157.2 (C(1)), 137.0 (C(5)), 133.1 (C(3)), 124.3 (C(4)), 124.3 (C(2)), 23.8 (C(6)), −5.6 (bd, C(7)). *cis* isomer *δ* 157.2 (C(1), 137.8 (C(5)), 133.1 (C(3)), 125.2 (C(4)), 124.9 (C(2)), 24.3 (C(6)), 1.94 (bd, C(7) & C(8)).
^{27}Al NMR (130.3 MHz, 25 ℃, d$_6$-benzene): *δ* 154.3.

Synthesis of [Me$_2$Al(6-MeO-2-py)]$_2$ (**3**): 2-Bromo-6-methoxypyridine (1.23 mL, 10 mmol) was dissolved in 20 mL of THF. The resultant solution was cooled to −78 ℃ and *n*-BuLi (1.6 M in hexanes, 6.25 mL, 10 mmol) was added dropwise; the reaction was stirred for 3 h. Subsequently, dimethylaluminium chloride (1 M in hexanes, 10 mL, 10 mmol) was added dropwise at −78 ℃. It is important to state here that dimethylaluminium chloride of a different molarity was used for the repeat reactions (0.9 M in heptanes, 11.1 mL). The reaction was allowed to warm overnight under a nitrogen atmosphere. The solvent from the resulting brown solution was removed *in vacuo* and replaced with 40 mL of toluene. The mixture was then filtered, and the filtrate was concentrated *in vacuo* until a precipitate was formed. The product was re-dissolved with gentle heating and refrigerated at 5 ℃ overnight to yield a yellowish-white crystalline product. The solvent was removed via cannula and the product was washed with pentane (5 mL); the product was then dried *in vacuo*. Yield 0.163 g, 12%.
^{1}H NMR (500 MHz, 25 ℃, d$_6$-benzene): *trans* isomer *δ* 7.68 (dd, 2H, H(3)), 7.02 (mult, 2H, H(4)), 5.64 (dd, 2H, H(2)), 3.06 (s, 6H, H(6)), −0.01 (s,12H, Me$_2$Al).
^{13}C NMR (126 MHz, 25 ℃, d$_6$-benzene): *δ* 163.9 (C(5)), 139.1 (C(1) & C(3)), 102.4 (C(2) & C(4)), 54.5 (C(6)), 0.93 (bd, C(7) & C(8)).
^{27}Al NMR (130.3 MHz, 25 ℃, d$_6$-benzene): *δ* 155.3.

Synthesis of [^{i}Bu$_2$Al(2-py)]$_2$ (**4**): 2-Bromopyridine (0.95 mL, 10 mmol) was dissolved in 20 mL of THF. The resultant solution was cooled to −78 ℃ and *n*-BuLi (1.6 M in hexanes, 6.25 mL, 10 mmol) was added dropwise; the reaction was stirred for 3 h. Subsequently, diisobutylaluminium chloride (0.8 M in hexanes, 12.5 mL, 10 mmol) was added dropwise at −78 ℃. The reaction was allowed to warm overnight under a nitrogen atmosphere. The solvent from the resulting brown solution was removed *in vacuo* and replaced with 40 mL of toluene. The mixture was then filtered, and the filtrate was concentrated *in vacuo* until a precipitate was formed. The product was re-dissolved with gentle heating and refrigerated at 5 ℃ overnight to yield a colourless crystalline product. The solvent was removed via cannula and the product was washed with pentane (5 mL); the product was then dried *in vacuo*. Yield 0.330 g, 24%.
^{1}H NMR (500.1 MHz, 25 ℃, d$_6$-benzene): *trans* isomer (35.5%) *δ* 8.30 (dt, 2H, H(5)), 7.91 (dt, 2H, H(2)), 7.03 (mult, 2H, H(3)), 6.62 (mult, 2H, H(4)), 1.84 (sept, 4H, H(7)/H(7')), 0.96 (d, 12H, H(8)), 0.93 (d, 12H, H(9)), 0.42 (d, 8H, H(6)/H(6')–Al). *cis* isomer (64.5%) *δ* 8.30 (dt, 2H, H(5)), 7.80 (dt, 2H, H(2)), 7.06 (mult, 2H, H(3)), 6.63 (mult, 2H, H(4)), 1.96 (sept, 2H, H(7)), 1.71 (sept, 2H, H(10)), 1.03 (d, 12H, H(8)), 0.87 (d, 12H, H(11)), 0.46 (d, 4H, H(6)–Al), 0.38 (d, 4H, H(10) –Al).
^{13}C NMR (126 MHz, 25 ℃, d$_6$-benzene): *trans* isomer *δ* 187.7 (C(1)), 146.3 (C(5)), 135.3 (C(3)), 134.9 (C(4)), 121.5 (C(2)), 28.3 (C(8)), 28.1 (C(9)), 27.3 (C(6)), 26.6 (C(7)). *cis* isomer *δ* 187.7 (C(1)), 145.3 (C(5)), 135.8 (C(3)), 135.1 (C(4)), 121.1 (C(2)), 28.6 (C(8)), 27.8 (C(9)), 27.3 (C(6)), 26.0 (C(7)).
^{27}Al NMR (130.3 MHz, 25 ℃, d$_6$-benzene): *δ* 145.1.

After leaving at room temperature for 11 days,
^{1}H NMR (500.1 MHz, 25 ℃, d$_6$-benzene): *trans* isomer (57.5%) *δ* 8.34 (dt, 2H, H(5)), 7.79 (dt, 2H, H(3)), 7.04 (mult, 2H, H(4)), 6.61 (mult, 2H, H(2)), 1.83 (sept, 4H, H(7)), 0.95 (d, 12H, H(8)), 0.93 (d, 12H, H(9)), 0.40 (d, 4H, H(6) –Al). *cis* isomer (42.5%) *δ* 8.29 (dt, 2H, H(5)), 7.90 (dt, 2H, H(3)), 7.02 (mult, 2H, H(4)), 6.62 (mult, 2H, H(2)), 1.95 (sept, 2H, H(7)), 1.70 (sept, 2H, H(7)), 1.02 (d, 12H, H(8)), 0.87 (d, 12H, H(9)), 0.45 (d, 2H, H(6) –Al), 0.37 (d, 4H, H(6') –Al).

After heating for 2 days at 60 ℃,
^{1}H NMR (500.1 MHz, 25 ℃, d$_6$-benzene): *trans* isomer *δ* 8.33 (dt, 2H, H(5)), 7.79 (dt, 2H, H(3)), 6.98 (mult, 2H, H(4)), 6.54 (mult, 2H, H(2)), 1.87 (sept, 4H, H(7)), 1.01 (d, 12H, H(8)), 0.98 (d, 12H, H(9)), 0.46 (d, 4H, H(6) –Al).

Synthesis of [^{i}Bu$_2$Al(6-Me-2-py)]$_2$ (**5**): 2-Bromo-6-methylpyridine (1.14 mL, 10 mmol) was dissolved in 20 mL of THF. The resultant solution was cooled to −78 ℃ and n-Buli (1.6 M in hexanes, 6.25 mL, 10 mmol) was added dropwise; the reaction was stirred for 3 h. Subsequently, diisobutylaluminium chloride (0.8 M in hexanes, 12.5 mL, 10 mmol) was added dropwise at −78 ℃. The reaction was allowed to warm overnight under a nitrogen atmosphere. The solvent from the resulting brown solution was removed in vacuo and replaced with 40 mL of toluene. The mixture was then filtered, and the filtrate was

concentrated in vacuo until a precipitate was formed. The product was re-dissolved with gentle heating and refrigerated at 5 ℃ overnight to yield a colourless crystalline product. The solvent was removed via cannula and the product was washed with pentane (5 mL); the product was then dried in vacuo. Yield 0.330 g, 24%. Although we can crystallise **5** at low temperature and obtain its single-crystal X-ray structure, the compound is a waxy solid at room temperature and repeated attempts to remove baseline impurities present in the NMR spectra were unsuccessful.

^{1}H NMR (500 MHz, 25 ℃, d$_6$-benzene): δ 7.86 (dd, 2H, H(3)), 6.98 (t, 2H, H(4)), 6.37 (dd, 2H, H(2)), 2.57 (s, 6H, H(6)), 1.81 (sept, 4H, H(8)), 1.03 (d, 12H, H(9)), 0.96 (d, 12H, H(10)), 0.50 (d, 4H, H(7) –Al).
^{13}C NMR (126 MHz, 25 ℃, d$_6$-benzene): δ 156.3 (C(5)), 135.6 (C(3)), 132.2 (C(1)), 123.2 (C(4)), 119.8 (C(2)), 28.4 (C(9) & C(10)), 27.9 (C(7)), 26.8 (C(8)), 22.8 (C(6)).
^{27}Al NMR (130.3 MHz, 25 ℃, d$_6$-benzene): δ 152.0.

Synthesis of [^{i}Bu$_2$Al(6-MeO-2py)]$_2$ (**6**): 2-Bromo-6-methoxypyridine (1.23 mL, 10 mmol) was dissolved in 20 mL of THF. The resultant solution was cooled to −78 ℃ and *n*-BuLi (1.6 M in hexanes, 6.25 mL, 10 mmol) was added dropwise; the reaction was stirred for 3 h. Subsequently, diisobutylaluminium chloride (0.8 M in hexanes, 12.5 mL, 10 mmol) was added dropwise at −78 ℃. The reaction was allowed to warm overnight under a nitrogen atmosphere. The solvent from the resulting brown solution was removed *in vacuo* and replaced with 40 mL of toluene. The mixture was then filtered, and the filtrate was concentrated *in vacuo* until a precipitate was formed. The product was re-dissolved with gentle heating and refrigerated at 5 ℃ overnight to yield a colourless crystalline product. The solvent was removed via cannula and the product was washed with pentane (5 mL); the product was then dried *in vacuo*. Yield 0.330 g, 24%.
^{1}H NMR (500 MHz, 25 ℃, d$_6$-benzene): *δ* 7.66 (dd, 2H, H(3)), 7.07 (t, 2H, H(4)), 5.72 (dd, 2H, H(2)), 3.15 (s, 6H, H(6)), 1.95 (sept, 4H, H(8)), 1.13 (d, 12H, H(9)), 1.08 (d, 12H, H(10)), 0.57 (d, 4H, H(7) –Al).
^{13}C NMR (126 MHz, 25 ℃, d$_6$-benzene): *δ* 163.8 (C(5)), 138.7 (C(3)), 128.2 (C(1)), 101.8 (C(2) & C(4)), 54.4 (C(6)), 28.7 (C(9) & C(10)), 28.1 (C(7)), 26.9 (C(8)).
^{27}Al NMR (130.3 MHz, 25 ℃, d$_6$-benzene): *δ* 150.8.

Demethylation of 3

A mixture of **3** (69.7 mg, 0.211 mmol) and [Ph$_3$C][B(C$_6$F$_5$)$_4$] (194 mg, 0.211 mmol) in toluene (10 mL) was stirred at room temperature for 3 h. A thin pale-yellow layer separated from the solution. Pentane (10 mL) was added to the solution and the mixture was stirred for 30 min. The oil was allowed to settle, and the upper layer was removed via syringe. Pentane (10 mL) was added to the oil and the mixture was stirred for 1 h. The solvent was then removed *in vacuo*; the resultant pale-yellow solid was rinsed with hexanes (3 x 5 mL) and dried under vacuum.^{1}H NMR (500 MHz, 25 ℃, d$_6$-benzene): *δ* 7.01–7.12 (mult, bd, 21H, H(2)–H(4) & 3 phenyl rings), 2.03 (s, 3H, H(9)), 1.32–1.35 (bd, 6H, H(6)), 1.24–1.26 (s, 3H, H(8)), 0.88 (s, 6H, H(7)).

NMR Spectroscopy

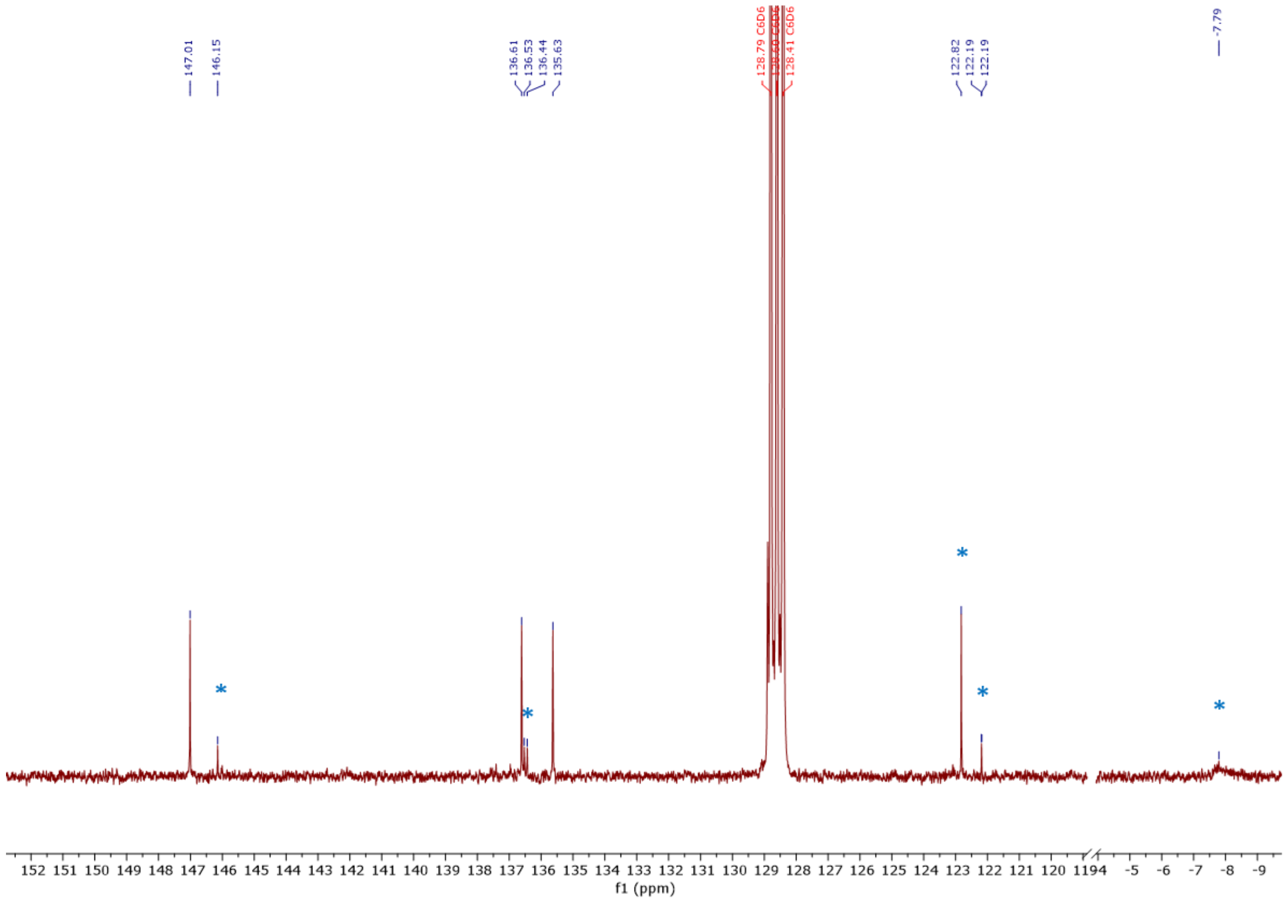

Figure S1. ^{13}C NMR spectrum (25 ℃, d_6-benzene, 126 MHz) of **1**. * is used to denote the signals arising from the *cis* isomer.

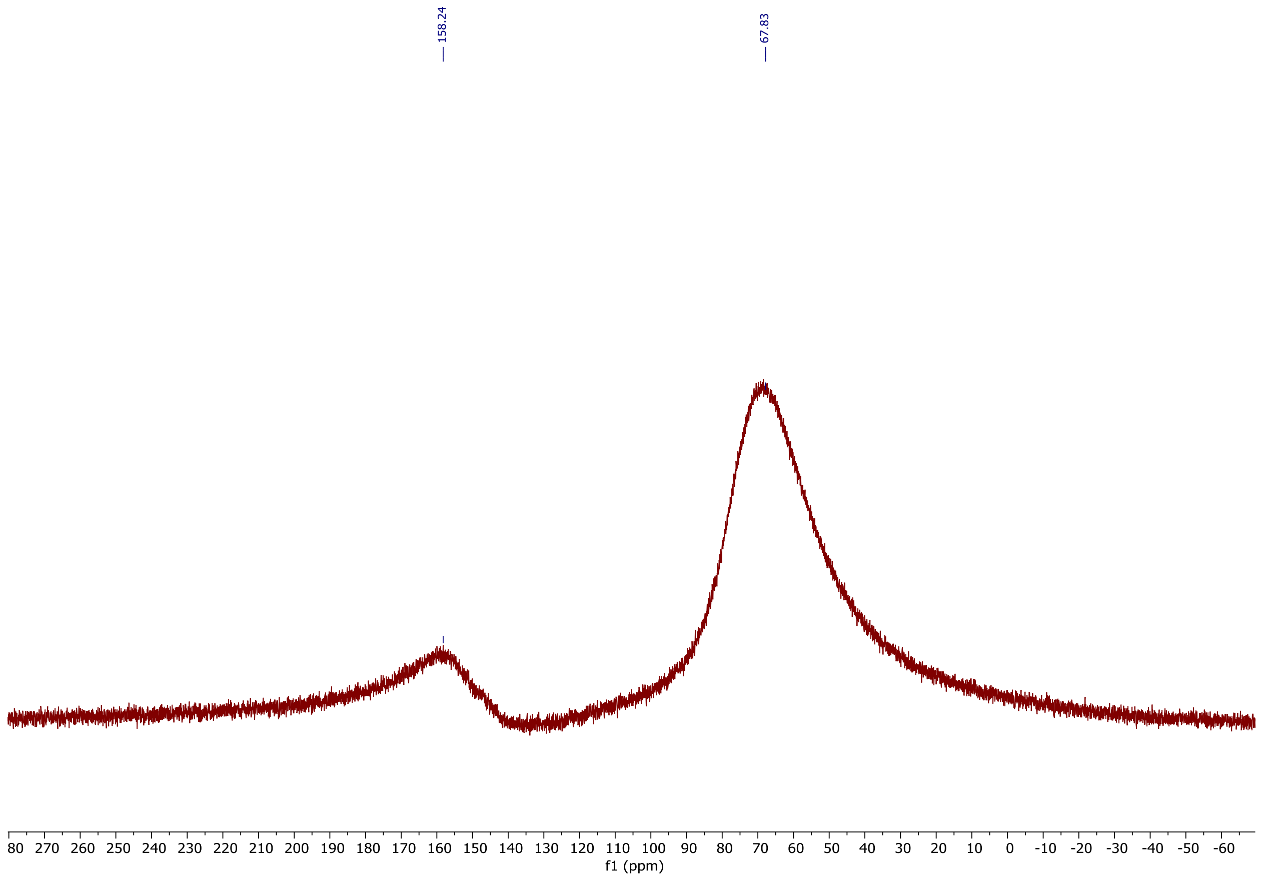

Figure S2. ^{27}Al NMR spectrum (25 ℃, d$_6$-benzene, 130.3 MHz) of **1**. The peak at *δ* 67.8 ppm is a background signal caused by the probe.

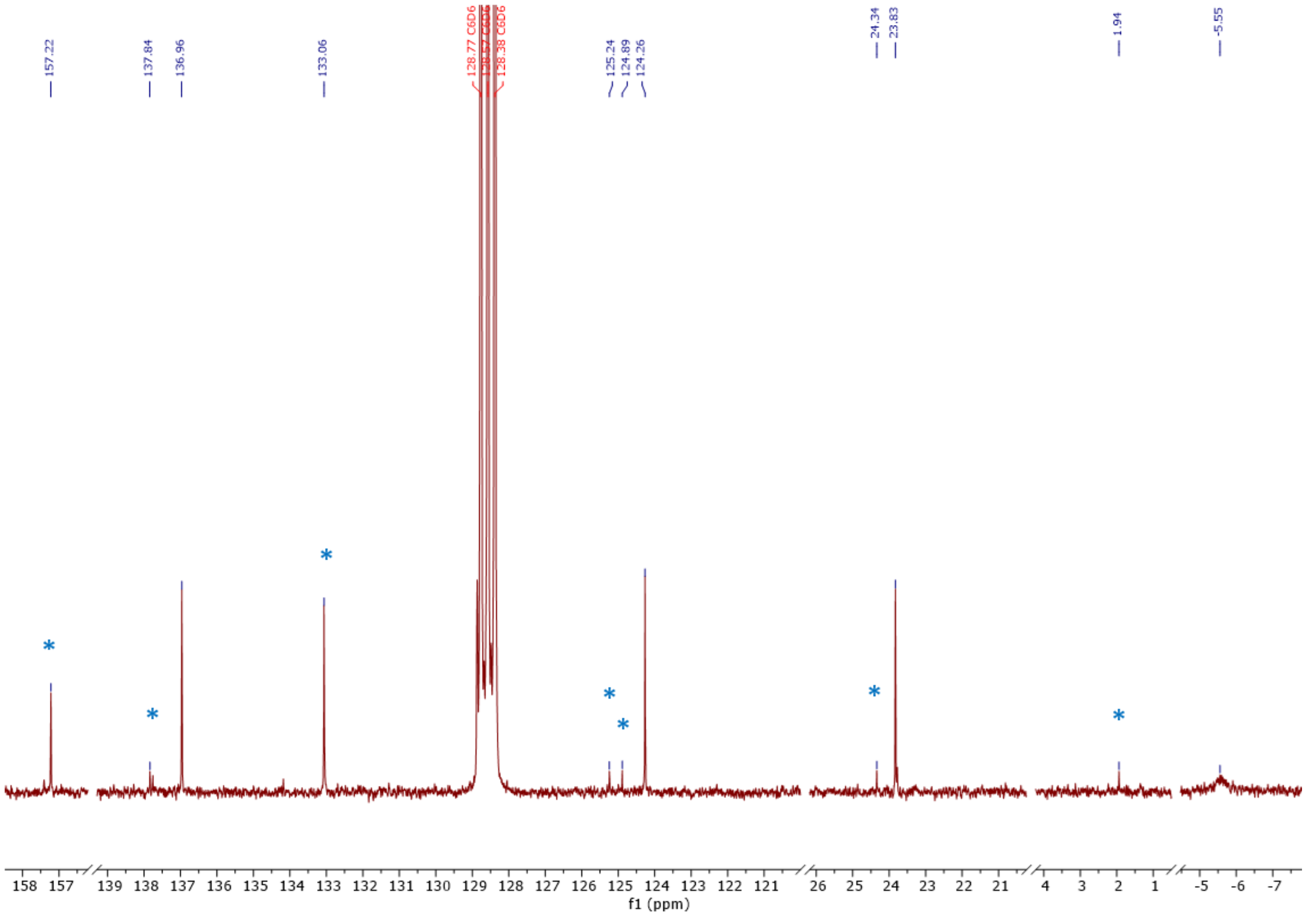

Figure S3. ^{13}C NMR spectrum (25 ℃, d_6-benzene, 126 MHz) of **2**. * is used to denote the signals arising from the *cis* isomer.

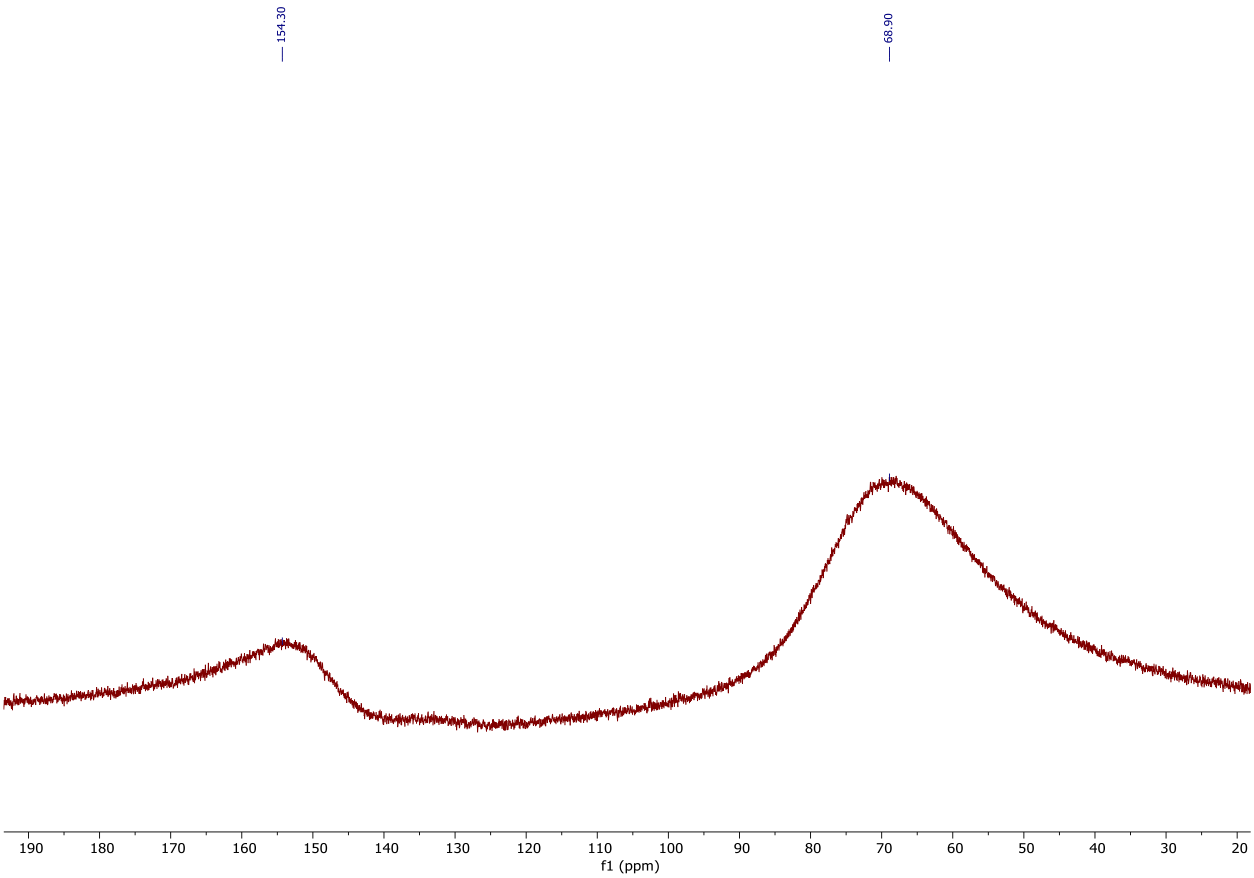

Figure S4. ^{27}Al NMR spectrum (25 ℃, d_6-benzene, 130.3 MHz) of **2**. The peak at δ 68.9 ppm is a background signal caused by the probe.

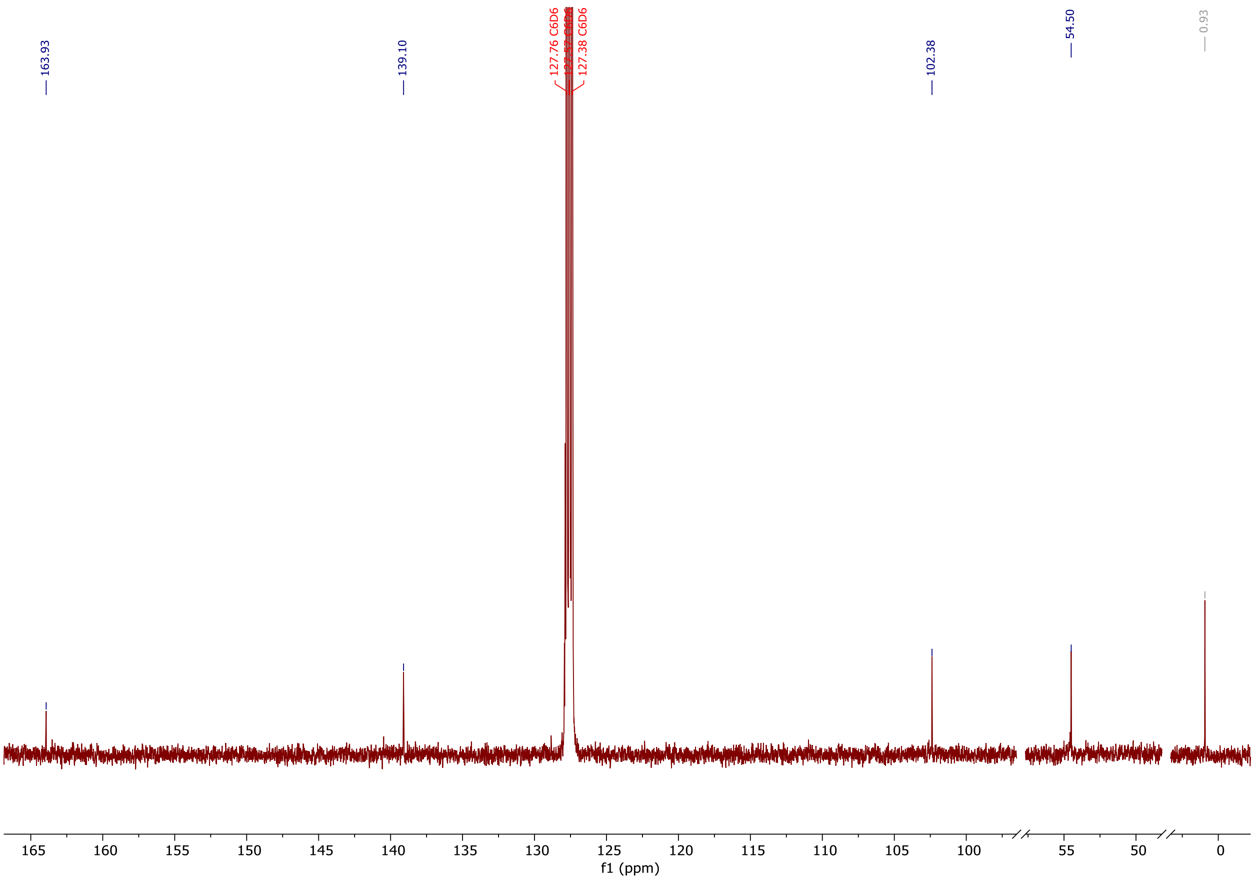

Figure S5. ^{13}C NMR spectrum (25 ℃, d_6-benzene, 126 MHz) of **3**.

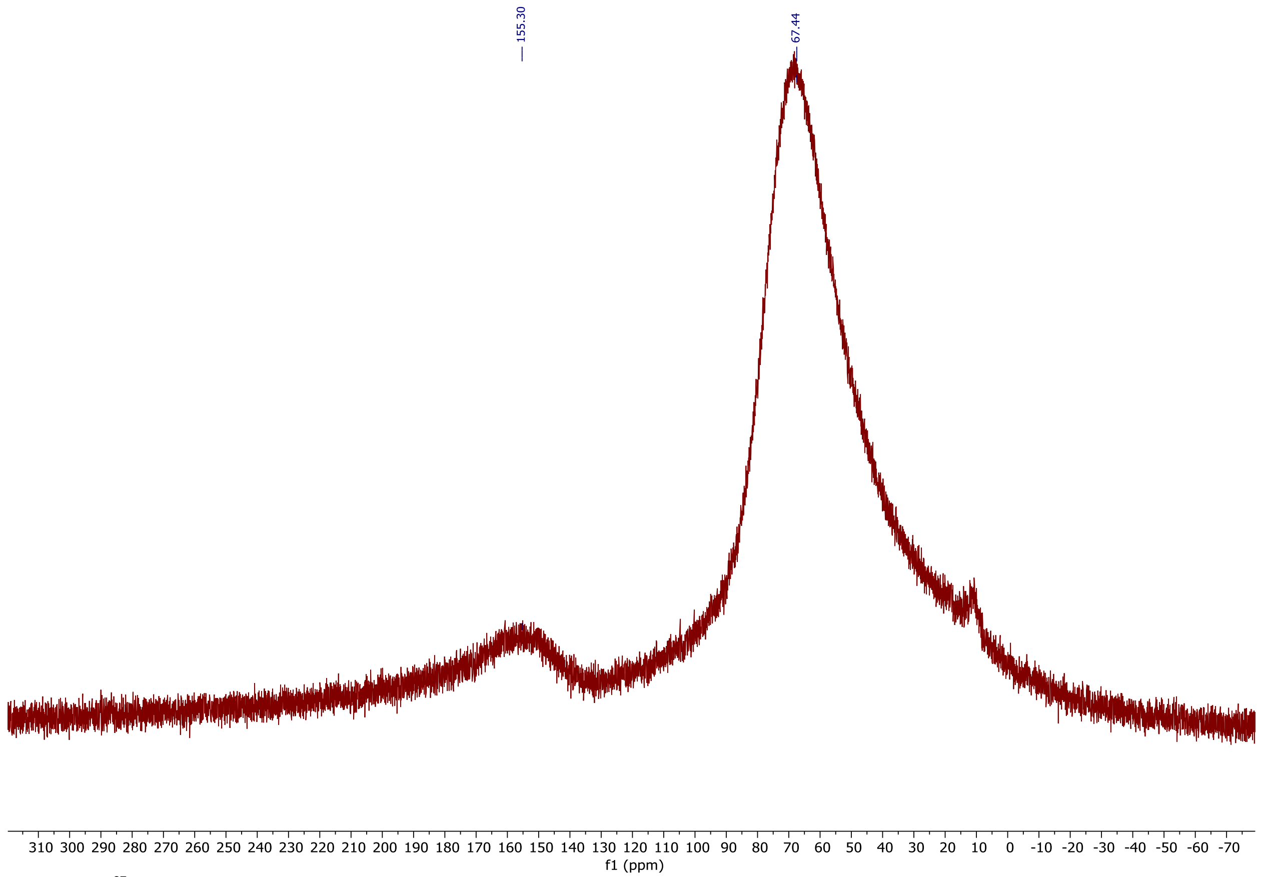

Figure S6. ^{27}Al NMR spectrum (25 ℃, d$_6$-benzene, 130.3 MHz) of **3**. The peak at *δ* 67.4 ppm is a background signal caused by the probe. The peak at ~10 ppm could not be accurately assigned.

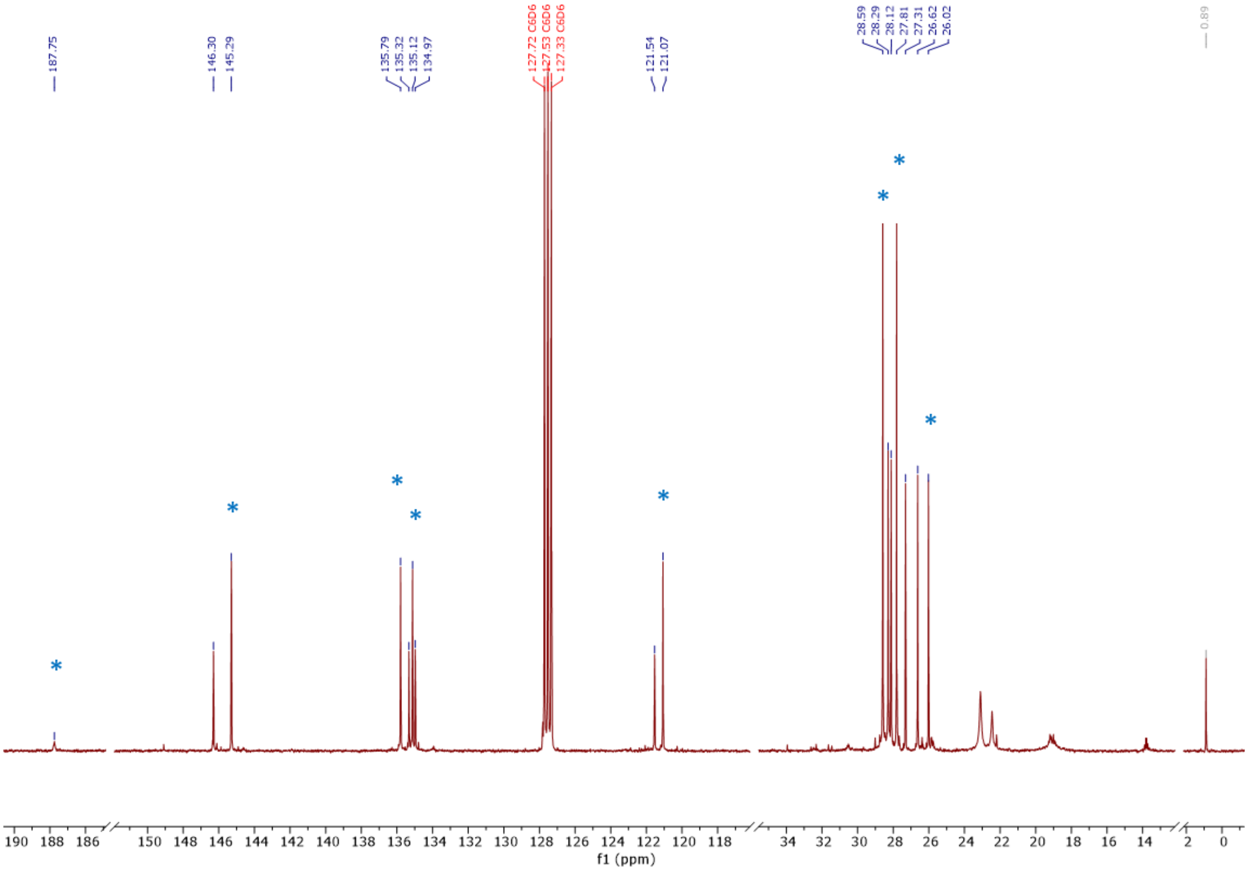

Figure S7. ^{13}C NMR spectrum (25 ℃, d_6-benzene, 126 MHz) of **4**. * is used to denote the signals arising from the *cis* isomer.

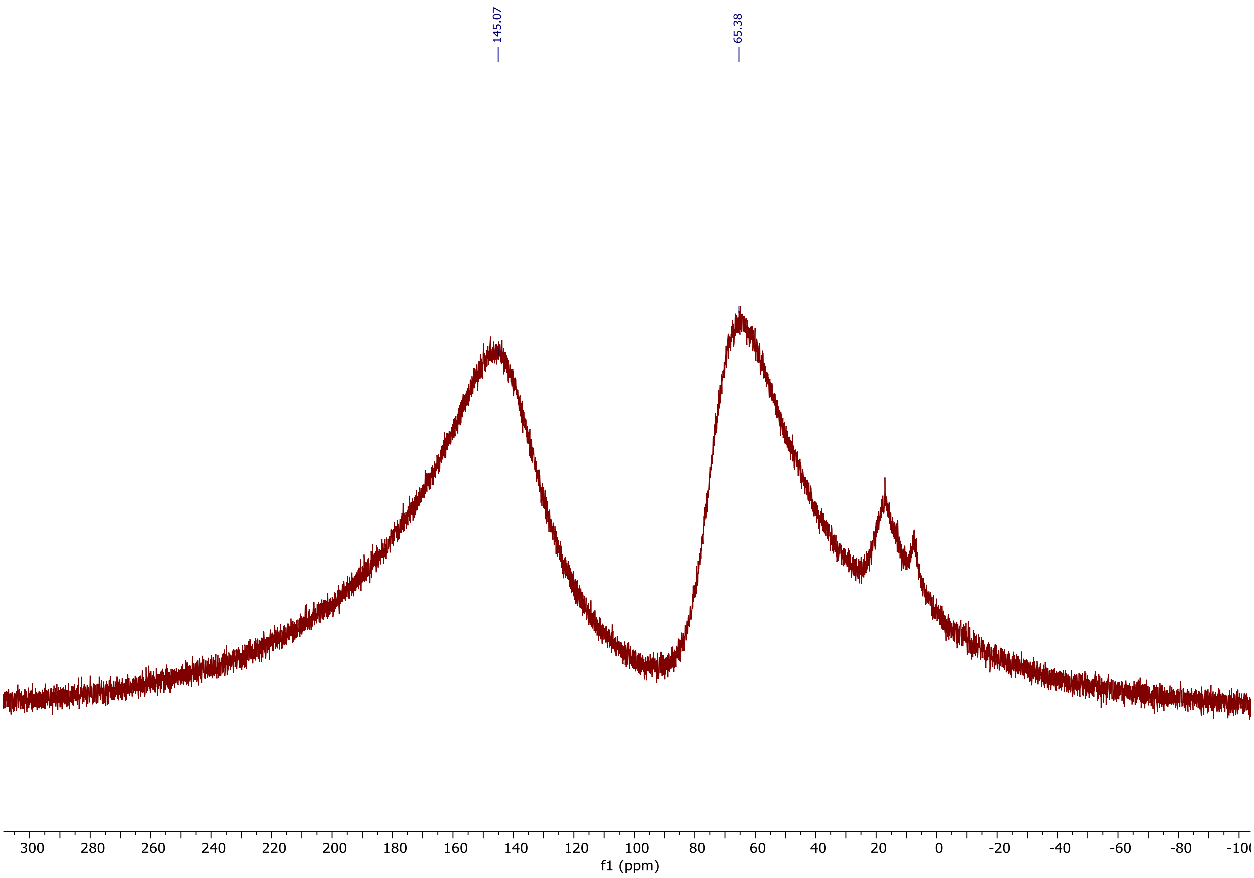

Figure S8. ^{27}Al NMR spectrum (25 ℃, d_6-benzene, 130.3 MHz) of **4**. The peak at δ 65.4 ppm is a background signal caused by the probe. The peaks at ~15 ppm and ~7 ppm could not be accurately assigned.

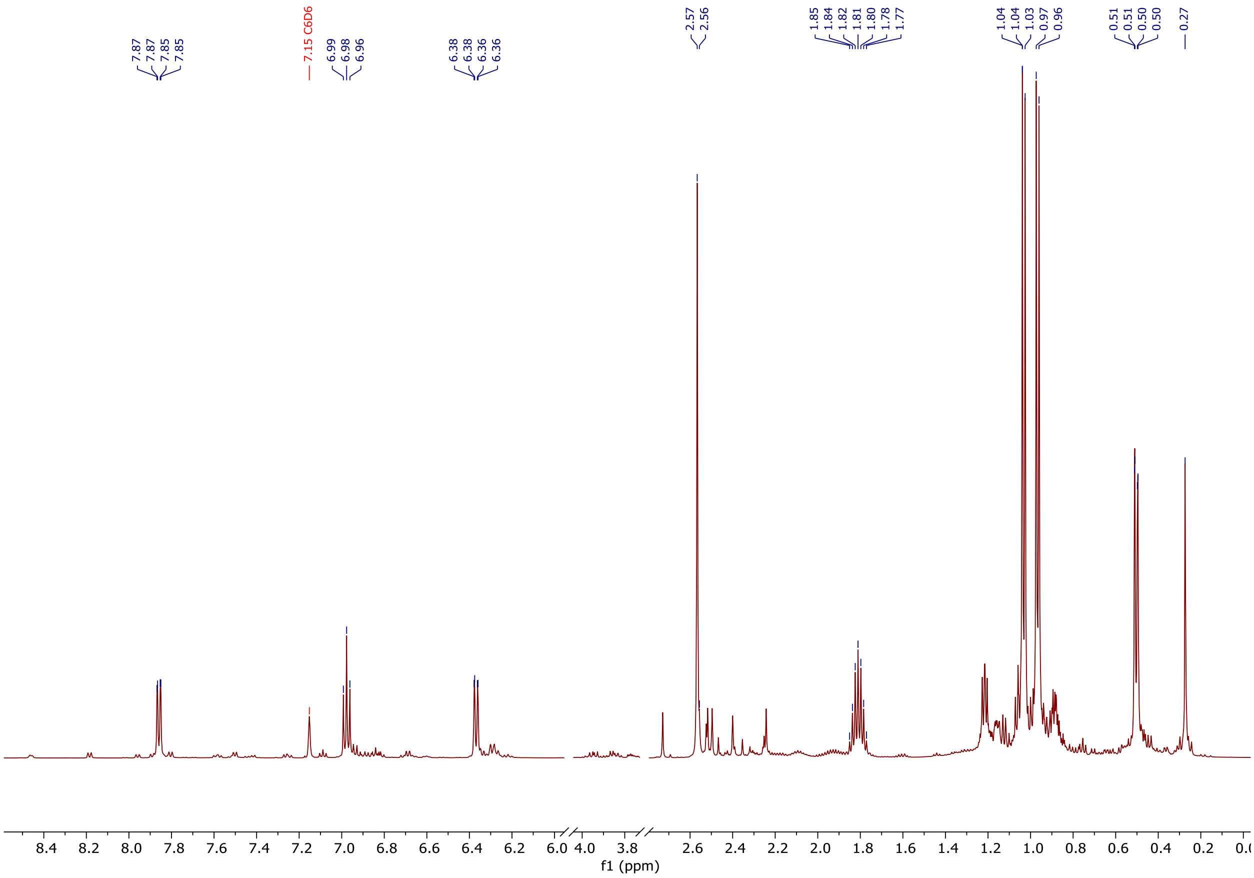

Figure S9. ^{1}H NMR spectrum (25 ℃, d$_6$-benzene, 500 MHz) of **5**. The peak at δ 0.27 ppm is vacuum grease.

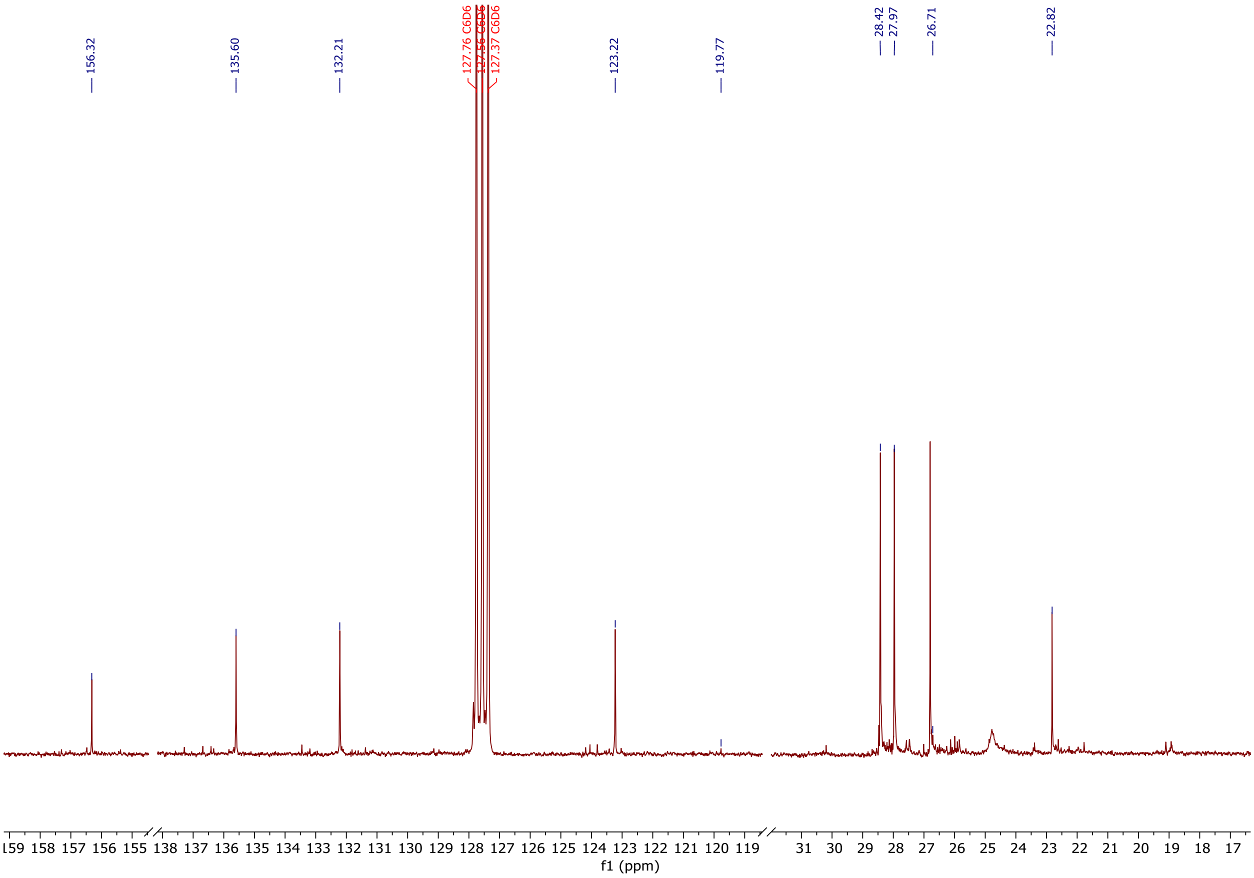

Figure S10. ^{13}C NMR spectrum (25 ℃, d_6-benzene, 126 MHz) of **5**.

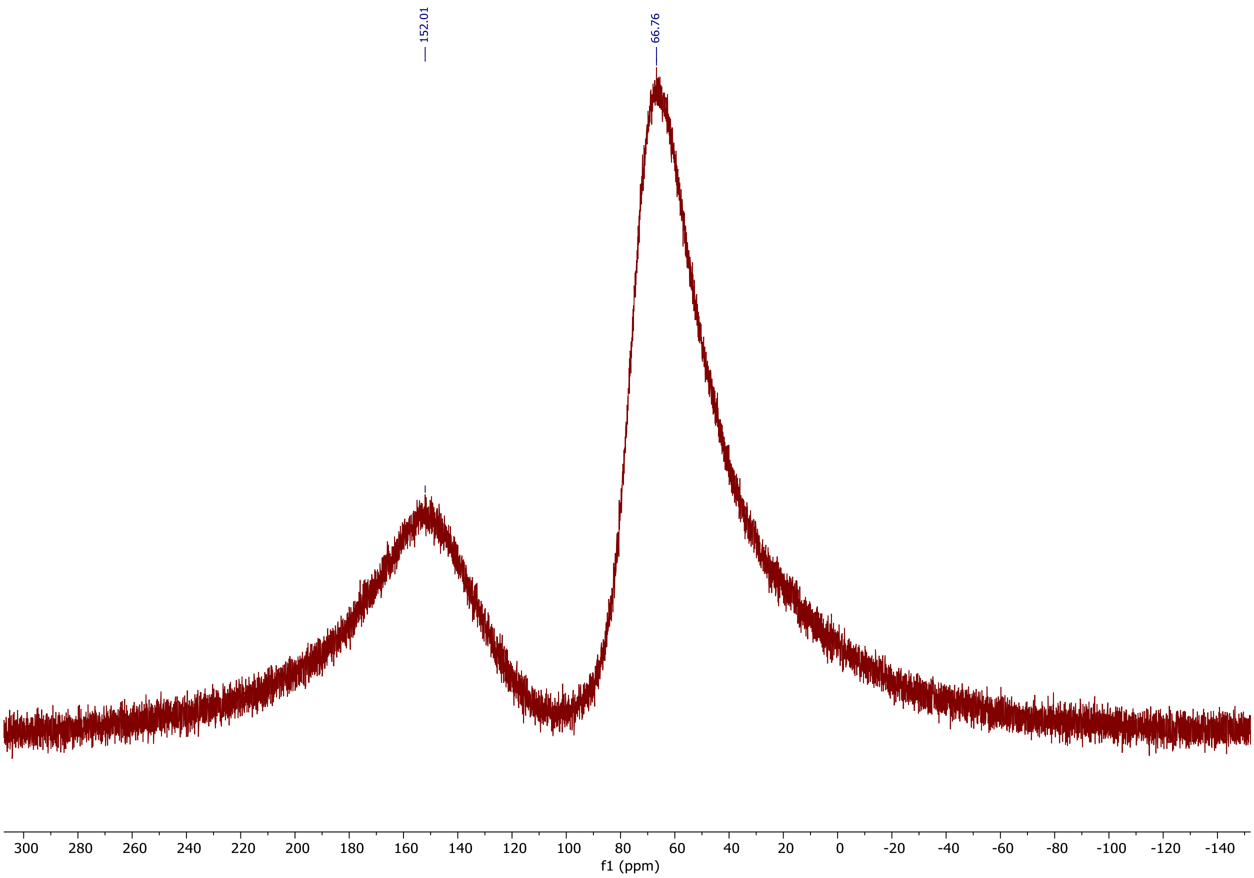

Figure S11. ^{27}Al NMR spectrum (25 ℃, d_6-benzene, 130.3 MHz) of **5**. The peak at δ 66.7 ppm is a background signal caused by the probe.

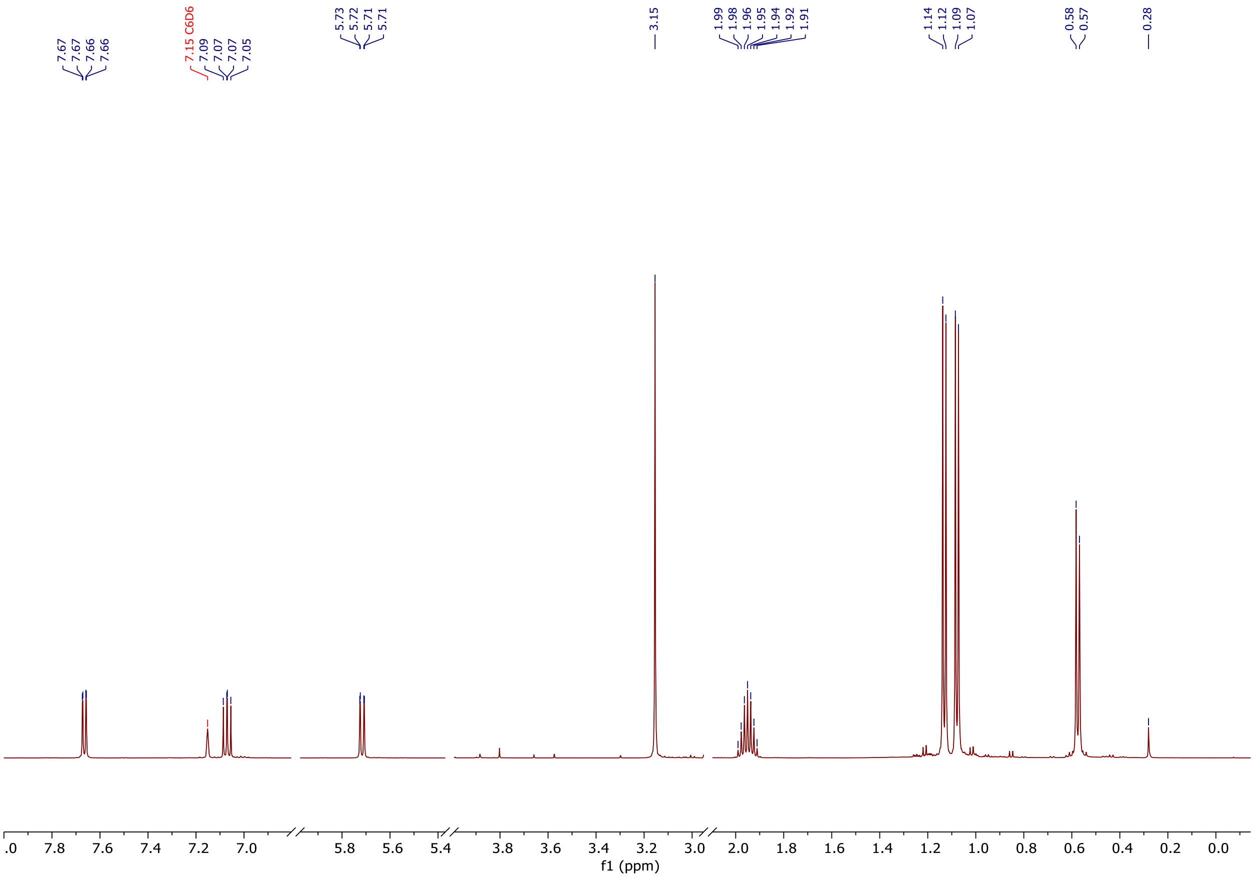

Figure S12. ^{1}H NMR spectrum (25 ℃, d_6-benzene, 500 MHz) of **6**. The peak at δ 0.28 ppm is vacuum grease.

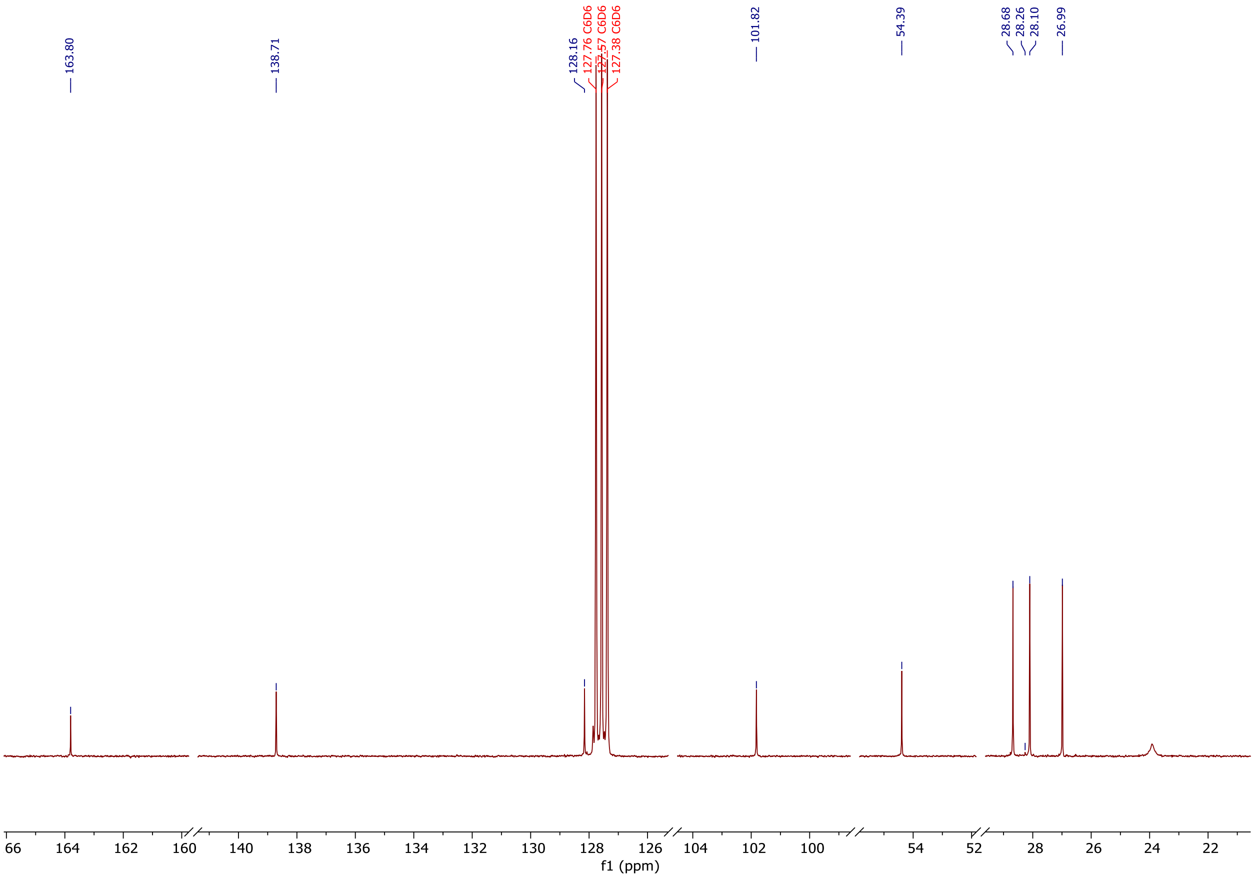

Figure S13. ^{13}C NMR spectrum (25 ℃, d_6-benzene, 126 MHz) of **6**.

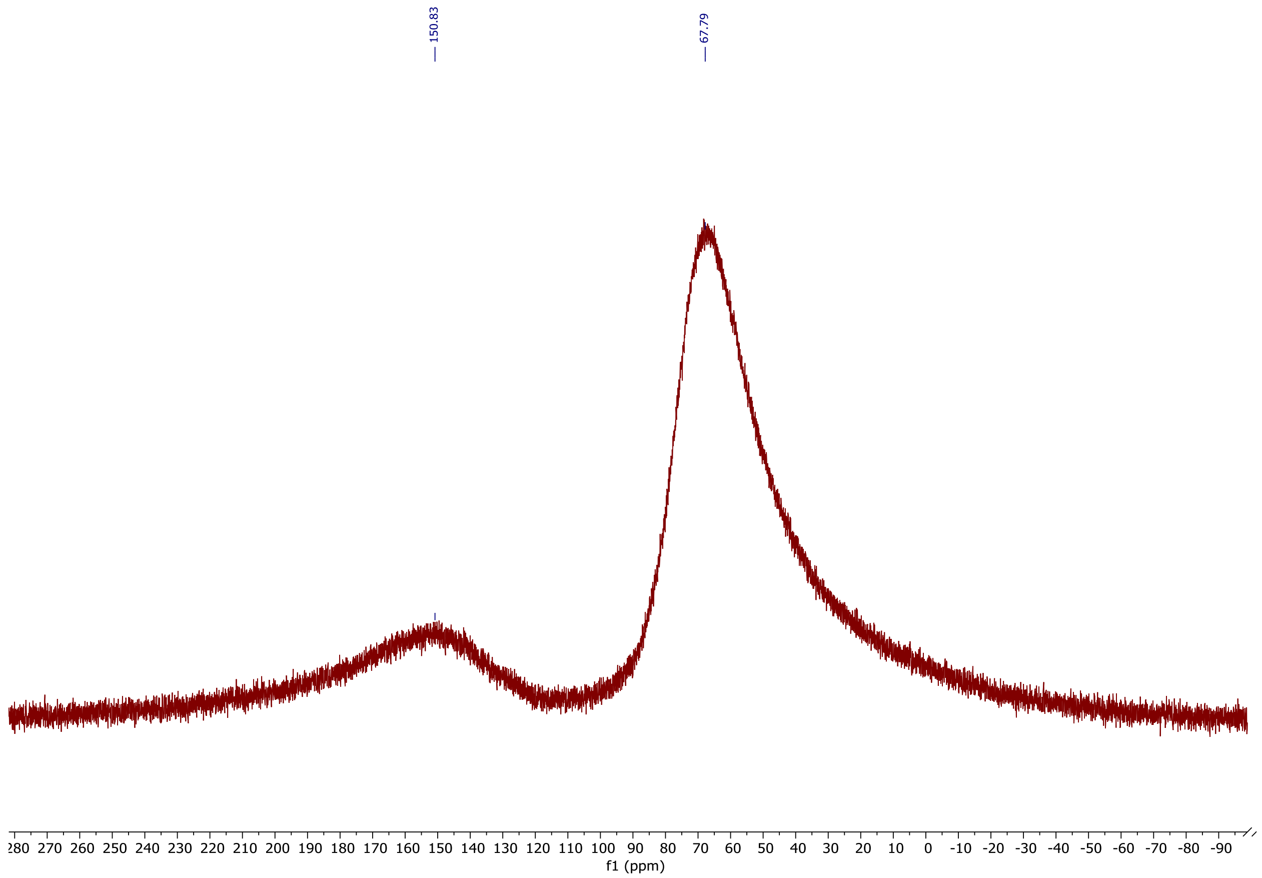

Figure S14. ^{27}Al NMR spectrum (25 ℃, d_6-benzene, 130.3 MHz) of **6**. The peak at δ 67.8 ppm is a background signal caused by the probe.

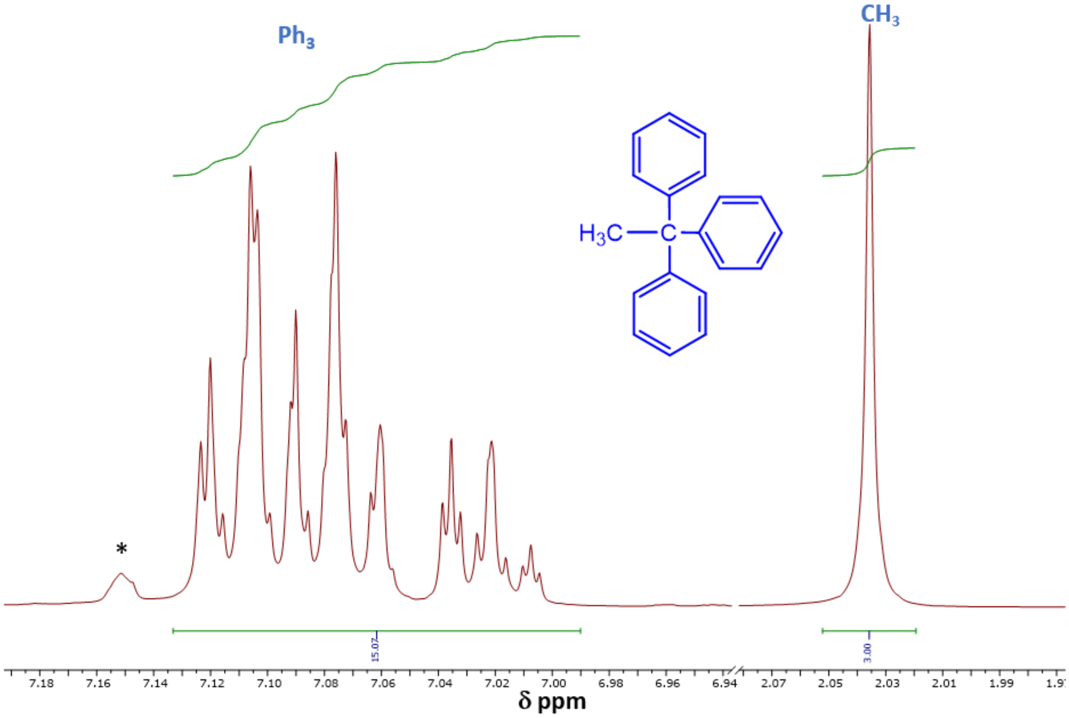

Figure S15. ^{1}H NMR (25 ℃, d_6-benzene, 500.1 MHz) spectrum of the yellow layer formed via the NMR-scale reaction of **1** with $[Ph_3C][B(C_6F_5)_4]$ (1:1)..

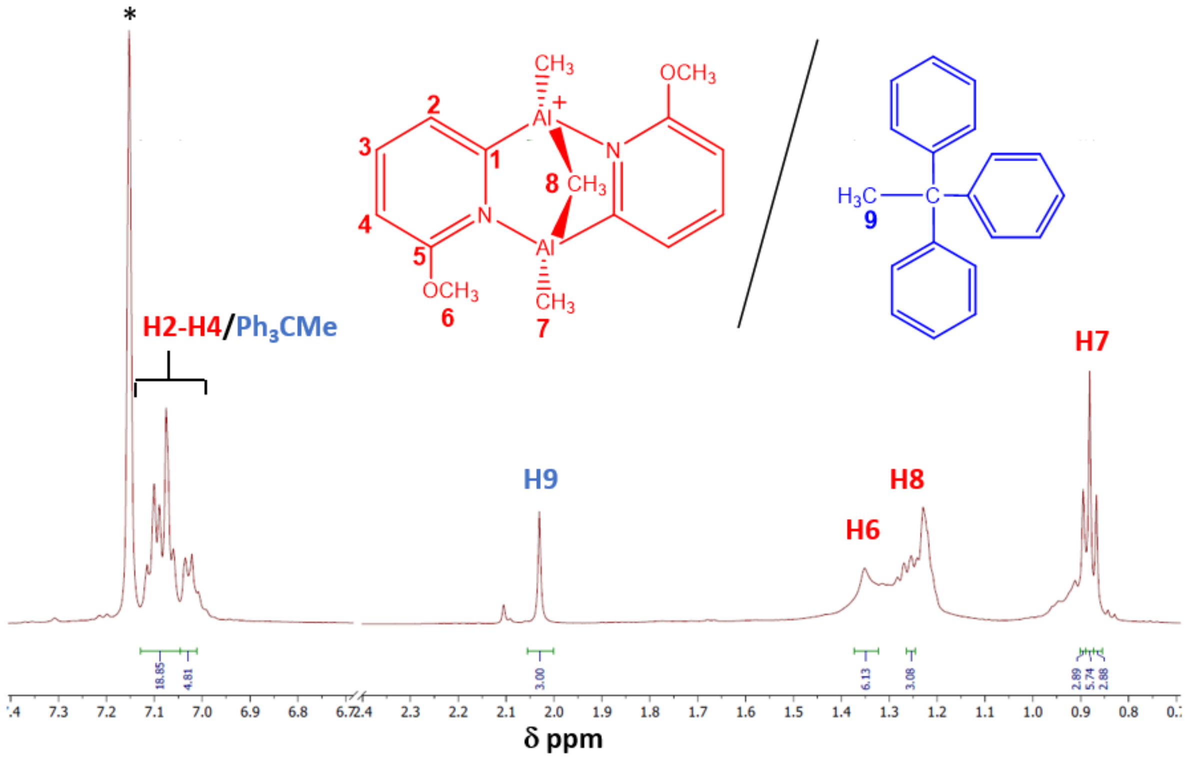

Figure S16. ^{1}H NMR (25 ℃, d_6-benzene, 500.1 MHz) spectrum of the reaction of **3** with $[Ph_3C][B(C_6F_5)_4]$ (1:1). The signals for H6 and H8 could not be resolved using variable-temperature ^{1}H NMR down to −50 ℃. * d_6-benzene.

X-ray Crystallography Data

Table S1. Crystallographic data and refinement details

	**2***	**3**	**4**	**5**	**6·(toluene)***
CCDC number	2290737	2290739	2290741	2290740	2290738
Cambridge data number	DW_B1_0418	DW_B2_0357	DW_B1_0487	DW_B1_0484	DW_B1_0489
Chemical formula	$C_{16}H_{24}Al_2N_2$	$C_{16}H_{24}Al_2N_2O_2$	$C_{26}H_{44}Al_2N_2$	$C_{28}H_{48}Al_2N_2$	$C_{35}H_{56}Al_2N_2O_2$
Moiety formula	$C_{16}H_{24}Al_2N_2$	$C_{16}H_{24}Al_2N_2O_2$	$C_{26}H_{44}Al_2N_2$	$C_{28}H_{48}Al_2N_2$	$C_{28}H_{48}Al_2N_2O_2$, C_7H_8
Formula weight	298.33	330.33	438.59	466.64	590.77
Temperature / K	180(2)	180(2)	220(2)	220(2)	220(2)
Crystal system	triclinic	triclinic	monoclinic	monoclinic	triclinic
Space group	P–1	P–1	$P2_1/c$	$P2_1/c$	P–1
a / Å	7.275(3)	7.5449(7)	8.8816(11)	9.6800(2)	9.0840(3)
b / Å	7.965(3)	8.3010(9)	17.784(3)	9.9564(2)	9.7211(3)
c / Å	9.237(3)	8.6197(10)	9.4629(10)	16.2198(4)	11.3181(4)
alpha / °	110.002(16)	75.765(7)	90	90	98.006(2)
beta / °	90.179(18)	87.326(7)	109.760(7)	106.6353(12)	99.517(2)
gamma / °	116.882(16)	63.308(7)	90	90	102.674(2)
Unit-cell volume / $Å^3$	440.6(3)	466.24(9)	1406.7(3)	1497.81(6)	945.55(6)
Z	1	1	2	2	1
Calc. density / g cm^{-3}	1.124	1.176	1.035	1.035	1.038
F(000)	160	176	480	512	322
Radiation type	Cu K□	Cu K□	Cu K□	Cu K□	Cu K□
Absorption coeff / mm^{-1}	1.416	1.469	1.017	0.979	0.907
Crystal size / mm^3	0.16 x 0.10 x 0.05	0.14 x 0.12 x 0.01	0.14 x 0.08 x 0.02	0.20 x 0.18 x 0.08	0.30 x 0.18 x 0.14
2-Theta range / °	10.38-108.44	10.62-133.14	10.58-133.63	15.30-133.32	10.19-133.36
Completeness to max 2□	0.984	0.913	0.996	0.978	0.990
No. of reflections measured	3636	8698	18600	43993	13902
No. of independent refl	1063	1568	2491	2597	3311
R(int)	0.0964	0.1049	0.1232	0.0439	0.0440
No. parameters / restraints	95 / 0	105 / 0	141 / 0	151 / 0	224 / 147
R1 (I > 2s(I))	0.0995	0.0678	0.0610	0.0457	0.0880
$wR(F^2)$ (all data)	0.2581	0.1832	0.1771	0.1264	0.2766
Goodness-of-fit on F^2	1.049	1.078	1.042	1.037	1.065
Largest diff. peak & hole / e $Å^{-3}$	0.429, -0.309	0.345, -0.282	0.425, -0.332	0.685, -0.223	0.452, -0.451

*It should be noted that the structure determinations of **2** and **6** are of very limited quality and have some problems with disorder and limited diffraction to higher angles. Nonetheless, their overall arrangements are unambiguous.

Structure refinement details

Diffraction images were integrated using *SAINT* in *APEX4*, and a multi-scan correction was applied using *SADABS* or *TWINABS*.[2] The final unit-cell parameters were refined against all reflections. Structures were solved using *SHELXT*[3] and refined using *SHELXL*.[4] H atoms were placed in idealised positions and allowed to ride during subsequent refinement. Several of the structures showed twinning and/or disorder, as described below.

<u>**Dimer 2**</u>: the lattice geometry approximates monoclinic *C* (a ≈ 14.21. b ≈ 7.28, c ≈ 9.24 Å, β ≈ 112.7°), and the crystal was twinned by 2-fold rotation around the pseudo-monoclinic axis. The diffraction pattern was indexed and integrated as a single component. Efforts to identify more than one component at the indexing/integration stage did not yield any satisfactory results. Initial structure solution and refinement of the structure in *P*–1 stalled with *R*1 ≈ 0.40. At this stage, *TWINROTMAT* in *PLATON*[5] identified the following potential twin laws:

[1] 2-fold rotation around real a axis: 1 0 0 / -1 -1 0 / 0 0 -1
[2] 2-fold rotation around reciprocal c axis: -1 0 0 / 0 -1 0 / 0.5 1 1
[3] combination of [1] and [2]: -1 0 0 / 1 1 0 / -0.5 -1 -1

Creating an HKLF-5 file with both twin laws [1] and [2] improved the refinement and yielded refined BASF values of *ca* 0.37 and 0.03. Hence, twin law [2] makes a minimal contribution and was dropped. The final refinement was carried out using the original HKLF-4 data with a standard TWIN instruction corresponding to twin law [1]. This gave a refined BASF = 0.391(7). This approach produced a better result than the HKLF-5 format produced using *TWINROTMAT*. The intensities showed a relatively rapid drop-off with diffraction angle, so the final data are restricted to a maximum resolution of 0.95 Å, with I/sigma(I) ≈ 1.8 in the highest resolution shell. The precision of the structure is limited accordingly.

Dimer 3: the diffraction pattern was integrated on the basis of three domains with substantial overlap, and the refinement is carried out as a 3-component twin using the HKLF-5 format. The applied twin laws correspond to 2-fold rotations around the real a axis and the reciprocal c axis (as for dimer **2**). The refined BASF values are 0.281(4) and 0.180(7), so all three components are significant and the HKLF-5 refinement produced the best result.

Dimer 4: without any substituent to fix the positions of C vs N in the pyridyl ring, the complex is modelled with disorder of the C and N atoms., as seen for dimer **1** (CSD: REWPEP). The site occupancies were refined for atoms C5/N1 and C5A/N1A, yielding 0.59(4):0.41(3).

Dimer 5: the diffraction pattern was integrated on the basis of two domains with substantial overlap, and an HKLF-5 format file was prepared from domain 1 and all composite reflections. The applied twin law corresponds to a 2-fold rotation around the real c axis. The refined BASF value is 0.360(2).

Dimer 6: the structure shows disorder of the ^{i}Bu groups, which was modelled with suitable geometrical restraints. The majority of the disordered atoms could be refined satisfactorily with anisotropic displacement parameters, restrained to resemble isotropic behaviour (ISOR in SHELX). Two atoms (C10 and C10A), belonging to different components of one disordered ^{i}Bu group, are sufficiently far apart to be split, but anisotropic refinement of the two sites produces heavily distorted displacement ellipsoids. These atoms are therefore refined isotropically, with U(iso) for both atoms constrained to a common refined value. The crystal structure includes one toluene solvent molecule per molecule of **6**, which is disordered across an inversion centre in space group P–1.

Displacement ellipsoid plots (50% probability for non-H atoms)

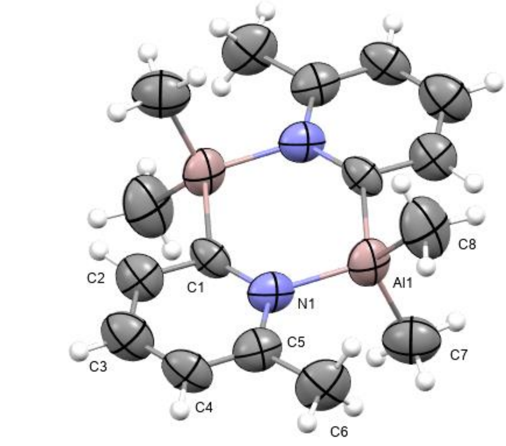

2 [molecule on inversion centre]

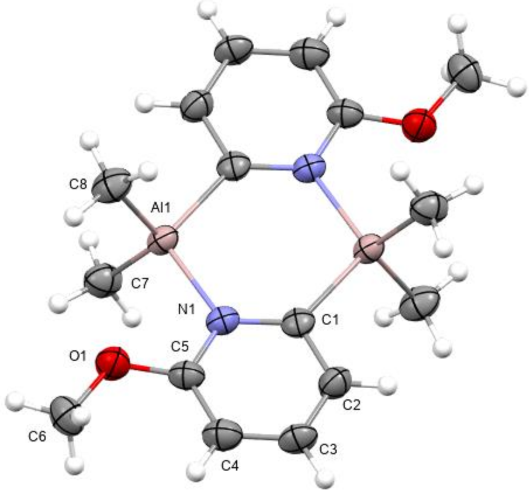

3 [molecule on inversion centre]

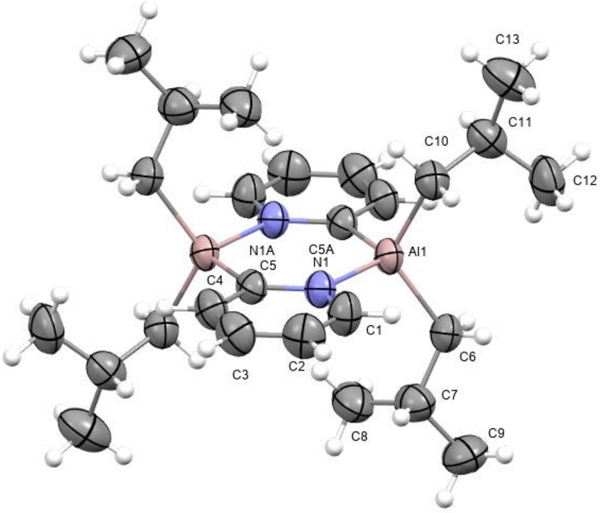

4 [molecule on inversion centre]

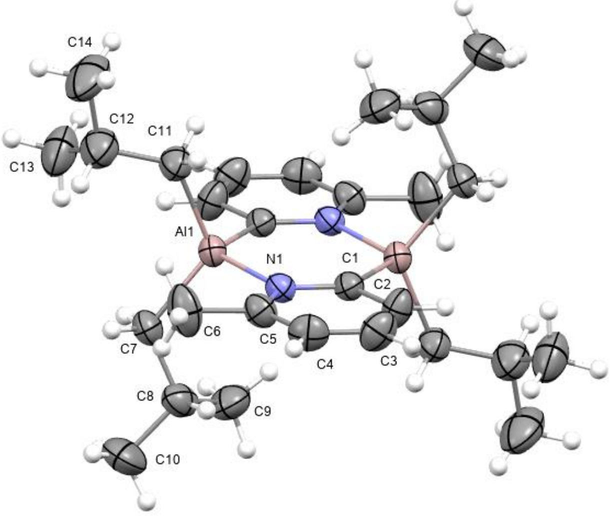

5 [molecule on inversion centre]

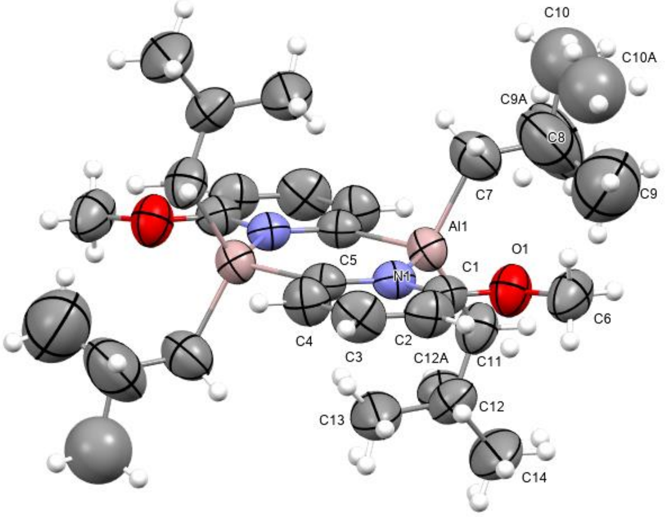

6 [molecule on inversion centre, toluene solvent molecule not shown]

Table S2. Selected bond distances and angles for 2–6

	**2**	**3**	**4**	**5**	**6**
Al—C_{py}	2.038(10)	2.043(5)	1.998(3)	2.030(2)	2.027(3)
Al—N_{py}	1.944(9)	1.973(4)	1.992(2)	1.9741(17)	1.977(2)
Al—C_R	1.924(14)	1.969(6)	1.983(3)	1.993(2)	1.974(3)
Al—C_R	1.970(12)	1.969(5)	1.977(3)	1.994(2)	1.994(4)
N_{py}—Al—C_{py}	110.4(4)	107.78(18)	108.55(10)	110.09(7)	107.12(11)
N_{py}—Al—C_R	108.9(5)	108.6(2)	104.17(12)	105.80(8)	105.89(15)
N_{py}—Al—C_R	109.3(5)	109.9(2)	108.41(13)	107.62(8)	110.37(13)
C_{py}—Al—C_R	103.8(5)	105.6(2)	108.03(12)	106.87(9)	106.25(17)
C_{py}—Al—C_R	107.2(5)	106.0(2)	109.45(13)	106.00(9)	105.88(13)
C_R—Al—C_R	117.2(7)	118.4(2)	117.89(13)	120.28(9)	120.61(17)

Computational study

Mechanistic study

Dimer **1** (R' = H, R = Me) was studied in detail to understand the mechanism of the isomerisation process. We have considered three different mechanism. The dissociative pathway is thermodynamically unfavourable with a $\Delta G_{dissociation}$ = 47.7 and 45.5 kcal mol^{-1} for the *trans* and *cis* dimer respectively. The details of the study on the concerted and stepwise mechanism are as presented below:

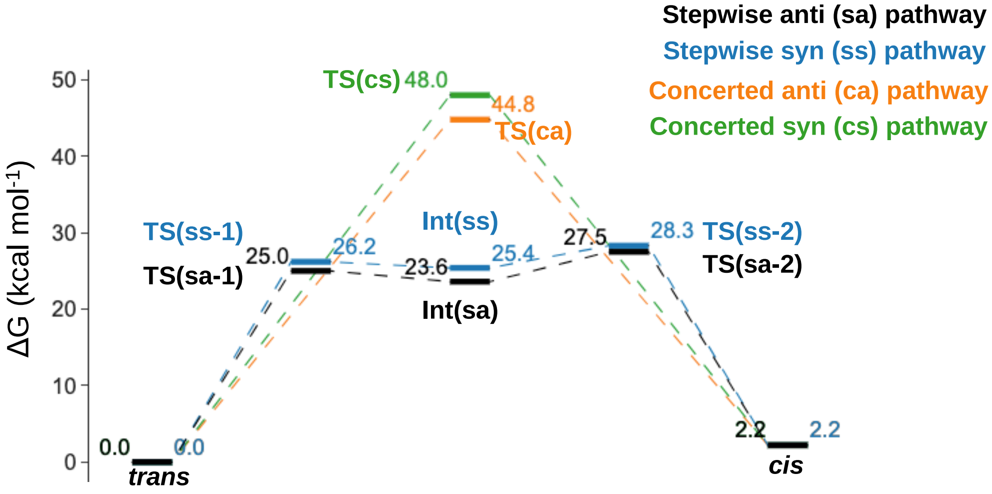

Figure S17. Energy profile of the concerted and stepwise mechanism (Dimer **1**; R' = H, R = Me).

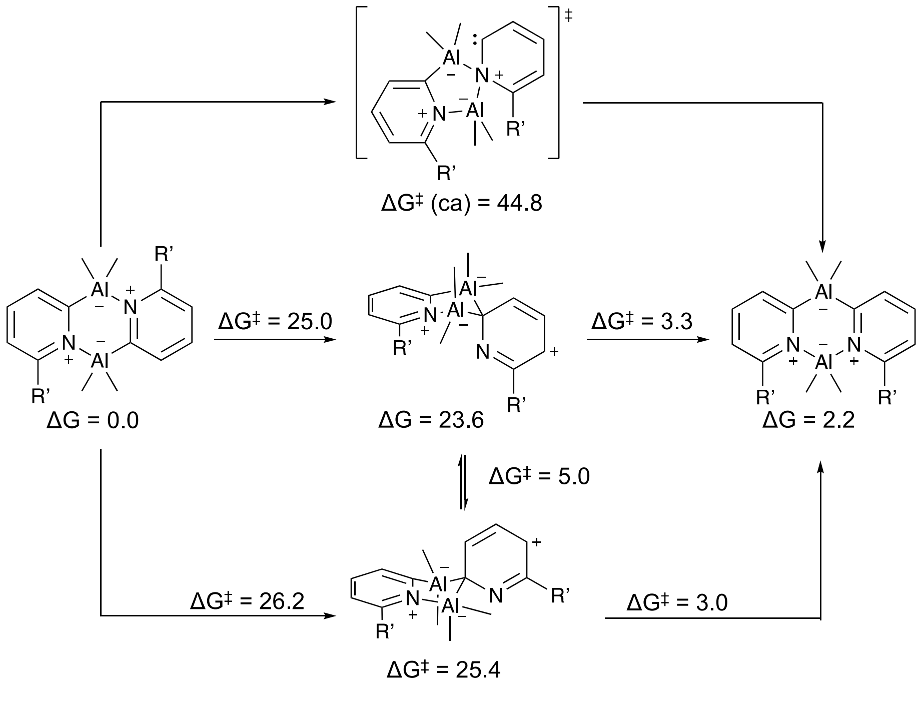

Figure S18. Diagrammatic illustration for the concerted and stepwise mechanism (Dimer **1**; R' = H, R = Me); unit: kcal mol^{-1}.

Key structures

Key ground state and transition state structures of dimer **1** isomerisation process are presented below:

1) Dimer **1**: R’ = H, R = Me

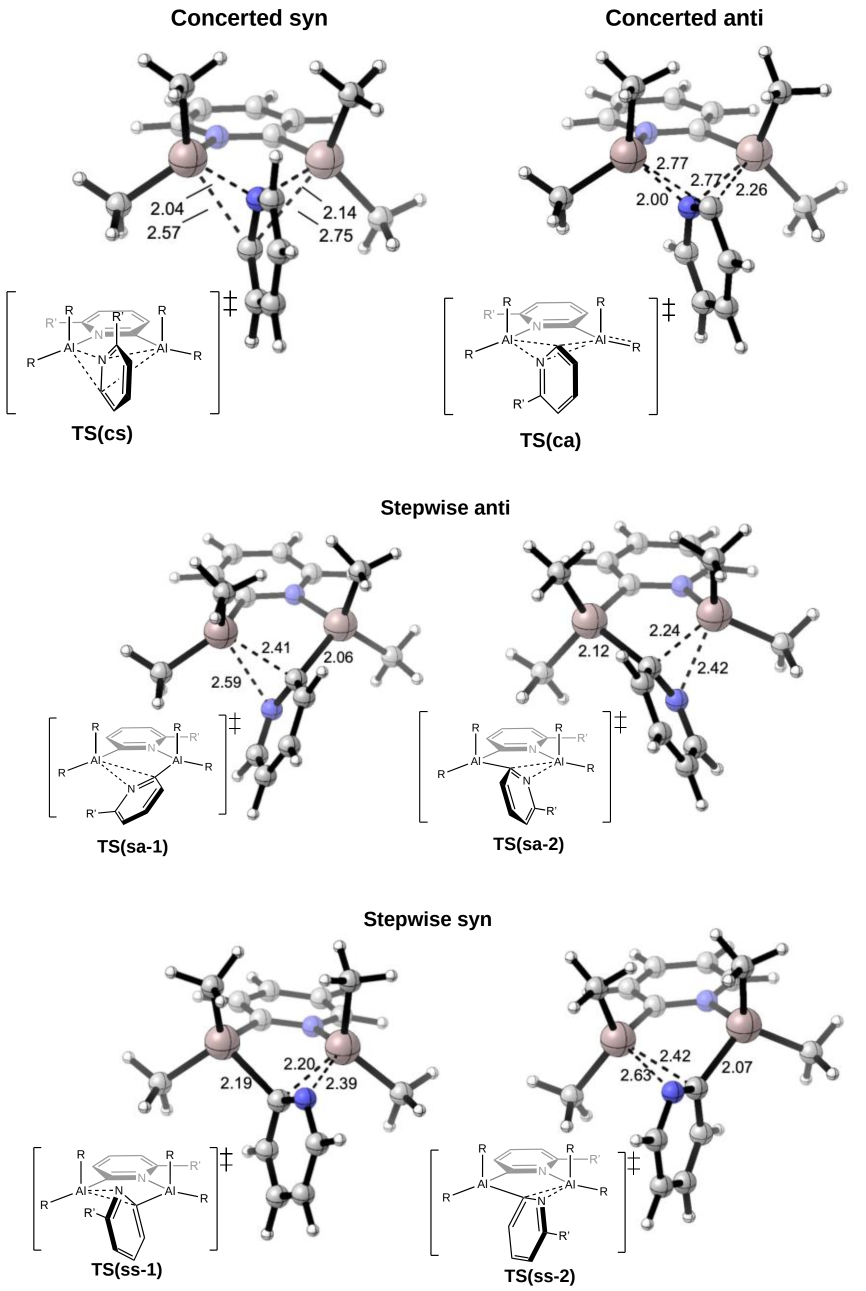

Figure S19. Key TS structures (Dimer **1**: R’ = H, R = Me).

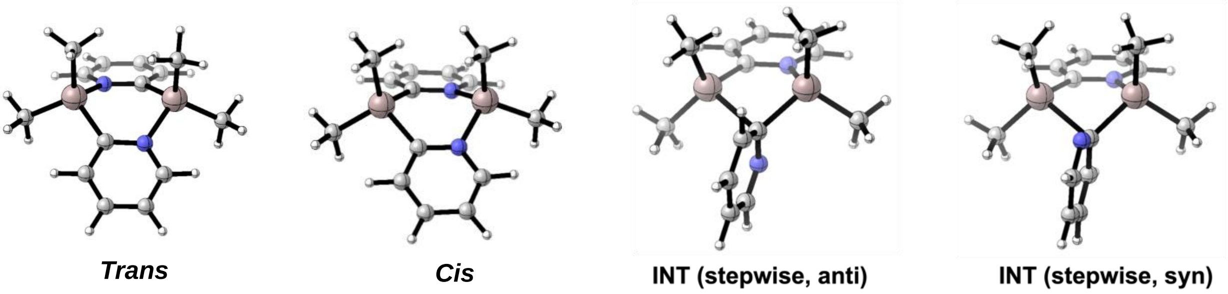

Figure S20. Key ground state structures (Dimer **1**: R’ = H, R = Me).

Based on results from the mechanistic study, the DFT calculations were performed to obtain the key ground state and transition state structures for the isomerisation of 2) dimer **2**: R' = Me, R = Me, 3) dimer **4**: R' = H, R = ^{i}Bu and 4) dimer **5**: R' = Me, R = ^{i}Bu systems. The energy profile and the structures of the key transition state are given below:

2) Dimer **2:** R' = Me, R = Me

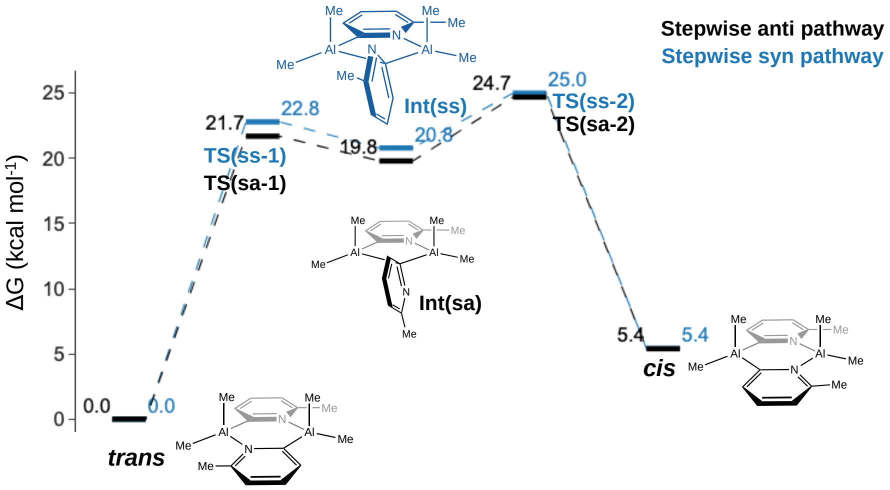

Figure S21. Energy profile of the stepwise pathways (Dimer **2:** R' = Me, R = Me).

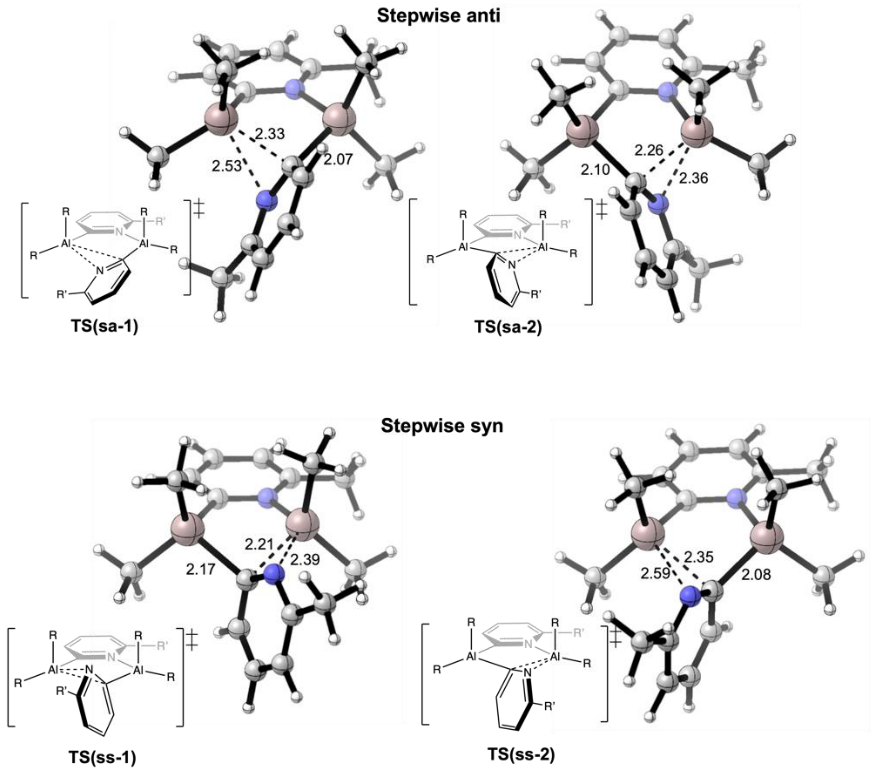

Figure S22. Key TS structures (Dimer **2:** R' = Me, R = Me).

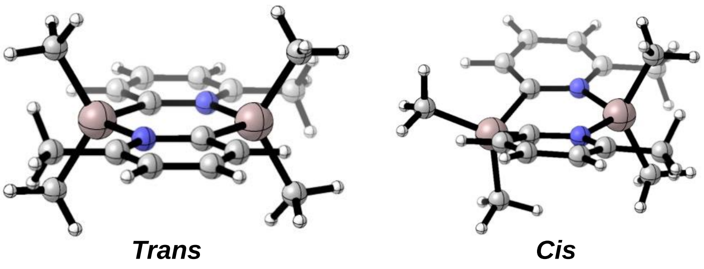

Figure S23. Key ground state structures (Dimer **2:** R' = Me, R = Me).

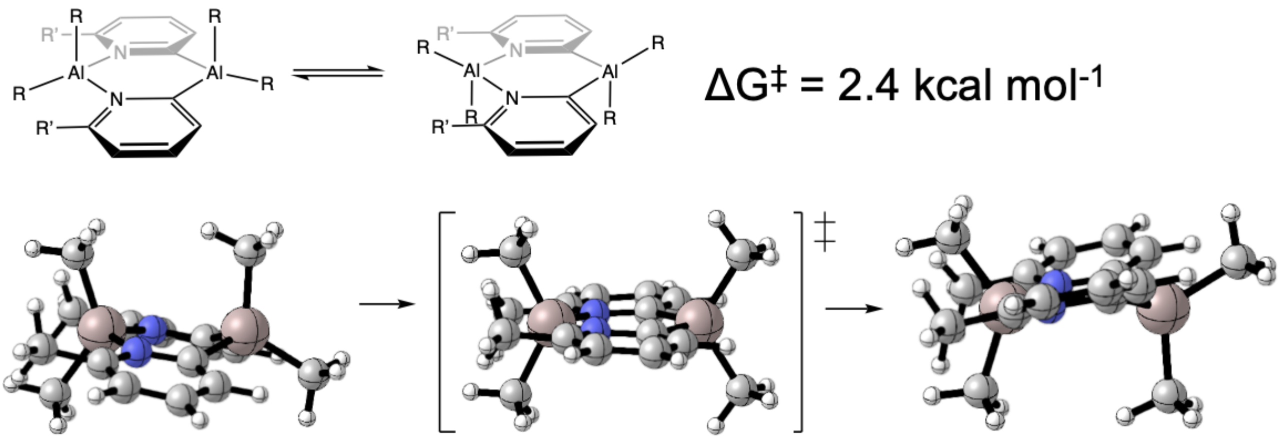

Figure S24. Inversion of the *cis* dimer via a planar transition state **2b** (R' = Me, R = Me)

3) Dimer **4**: R' = H, R = ^{i}Bu

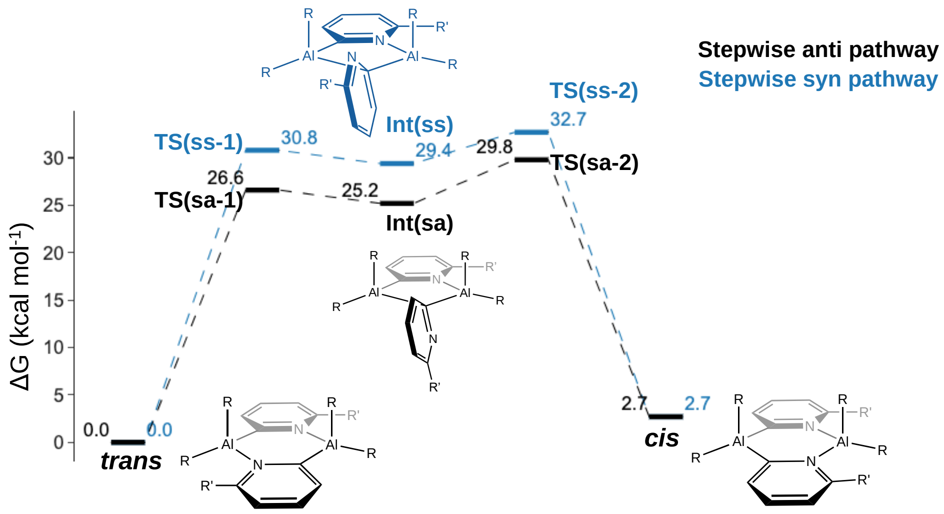

Figure S25. Energy profile of the stepwise pathways (Dimer **4**: R' = H, R = ^{i}Bu).

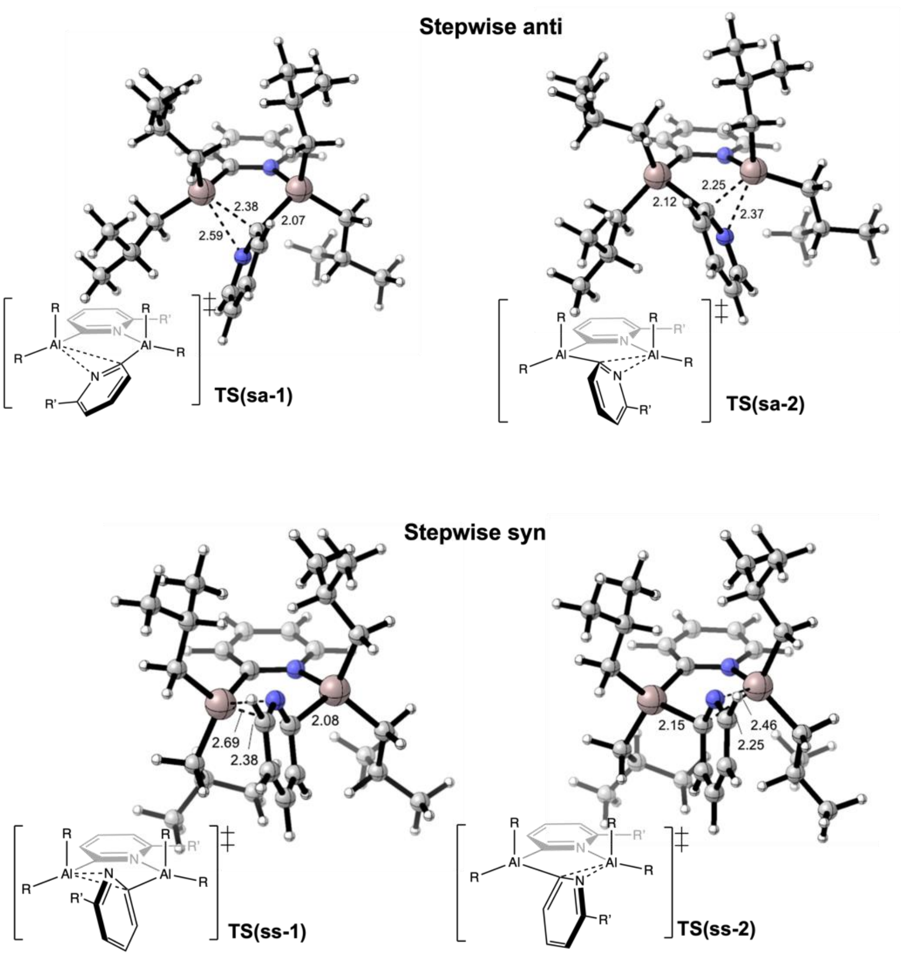

Figure S26. Key TS structures (Dimer **4**: R' = H, R = ^{i}Bu).

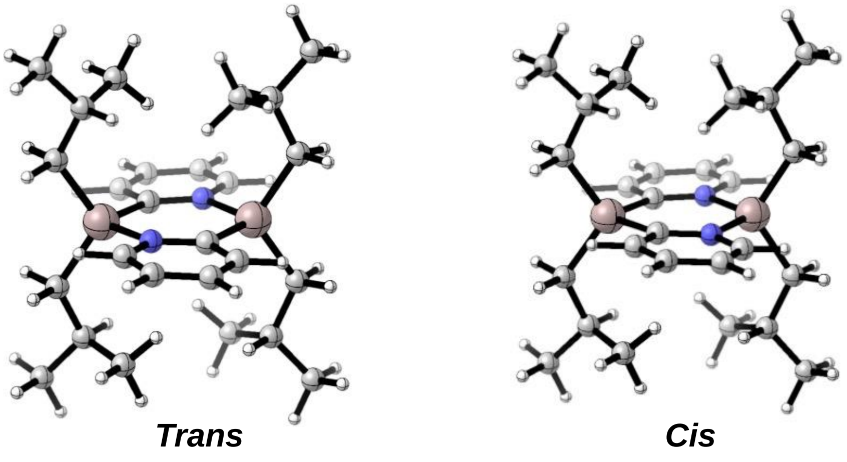

Figure S27. Key ground state structures (Dimer **4**: R' = H, R = ^{i}Bu).

4) Dimer **5**: R’ = Me, R = ^{i}Bu

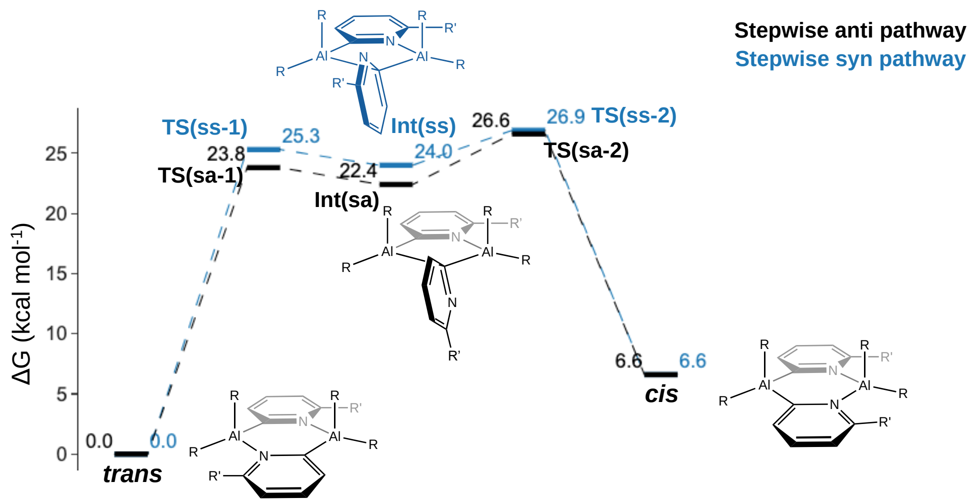

Figure S28. Energy profile of the stepwise pathways (Dimer **5**: R’ = Me, R = ^{i}Bu).

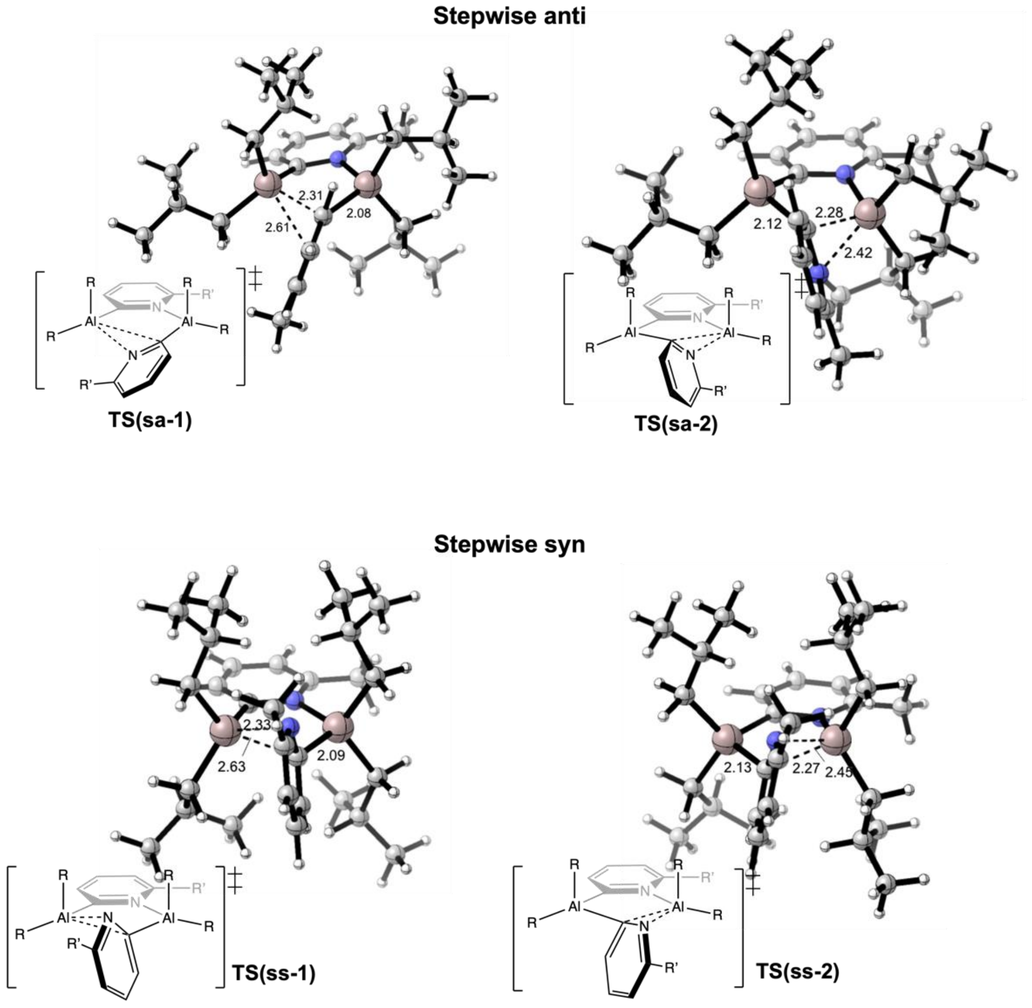

Figure S29. Key TS structures (Dimer **5**: R’ = Me, R = ^{i}Bu).

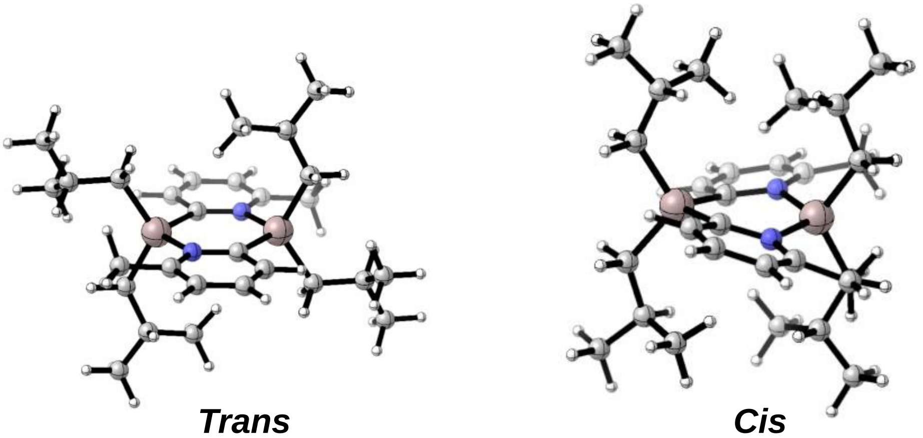

Trans ***Cis***

Figure S30. Key ground structures (Dimer **5**: R' = Me, R = ^{i}Bu).

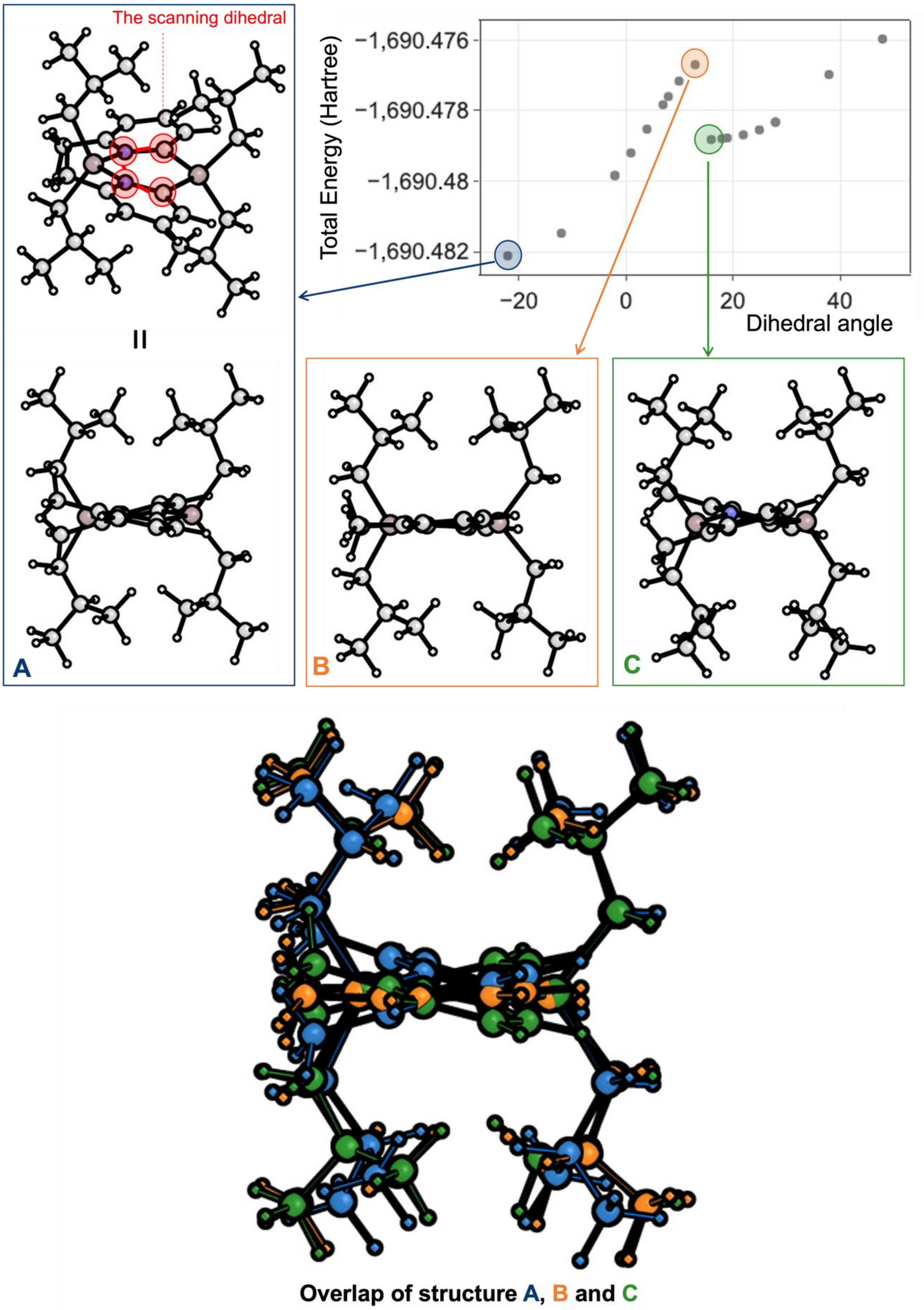

Figure S31. Investigating and searching for the ring flipping transition state (TS) for dimer 5.[15]

We estimate the barrier via a dihedral angle scan calculation. Structure A corresponds to the lowest energy group state complex 5. Structure B adopts a planar ring conformation and corresponds to the structure at the maximum of the total energy vs. dihedral angle plot in the figure. The energy difference between A and B is 3.4 kcal mol^{-1} (at the B3LPY-D3/6-31g(d) level of theory). We expect that this an overestimate for the energy barrier of the actual TS structure. We conclude that the barrier for flipping this ring is so low that it will be fast on the NMR timescale.

Computational Data
The structural information is available in the Cambridge Apollo Repository: https://doi.org/10.17863/CAM.99774

The key structures are included in the 'SI_key_structure_orgAl' folder as opt+freq or opt Gaussian calculation output files. The optimisations were conducted at the B3LYP-D3/6-31G(d) level of theory. The filename should be self-explanatory.

Directory tree:

```
SI_key_structure_orgAl/
├─ methylation/
│   ├─ Al_P_bonds_cis_MeH.out
│   ├─ Al_P_bonds_cis_MeMe.out
│   ├─ Al_P_bonds_trans_MeH.out
│   └─ Al_P_bonds_trans_MeMe.out
├─ R'=H_R=iBu/
│   ├─ iBuH_cis.out
│   ├─ iBuH_sa_int.out
│   ├─ iBuH_sa_TS1.out
│   ├─ iBuH_sa_TS2.out
│   ├─ iBuH_ss_int.out
│   ├─ iBuH_ss_TS1.out
│   ├─ iBuH_ss_TS2.out
│   └─ iBuH_trans.out
├─ R'=H_R=Me/
│   ├─ MeH_ca_TS_3.out
│   ├─ MeH_cis.out
│   ├─ MeMe_cis_ring_flipping_TS.out
│   ├─ MeH_cs_TS1.out
│   ├─ MeH_sa_int.out
│   ├─ MeH_sa_TS1.out
│   ├─ MeH_sa_TS2.out
│   ├─ MeH_ss_int.out
│   ├─ MeH_ss_TS1.out
│   ├─ MeH_ss_TS2.out
│   └─ MeH_trans.out
├─ R'=Me_R=iBu/
│   ├─ iBuMe_cis_sic2_1.out
│   ├─ iBuMe_sa_int.out
│   ├─ iBuMe_sa_TS1_2.out
│   ├─ iBuMe_sa_TS2_2.out
│   ├─ iBuMe_ss_int.out
│   ├─ iBuMe_ss_TS1.out
│   ├─ iBuMe_ss_TS2.out
│   └─ iBuMe_trans.out
└─ R'=Me_R=Me/
    ├─ MeMe_cis.out
    ├─ MeMe_sa_int.out
    ├─ MeMe_sa_TS1.out
    ├─ MeMe_sa_TS2.out
    ├─ MeMe_ss_int.out
    ├─ MeMe_ss_Me_TS1.out
    ├─ MeMe_ss_Me_TS2.out
    └─ MeMe_trans.out
```

References

1 F. García, A. D. Hopkins, R. A. Kowenicki, M. McPartlin, J. S. Silvia, J. M. Rawson, M. C. Rogers and D. S. Wright, *Chem. Commun.*, 2007, 586–588.

2

3 G. M. Sheldrick, *Acta Crystallogr A*, 2015, **71**, 3–8.

4 G. M. Sheldrick, *Acta Crystallogr C Struct Chem*, 2015, **71**, 3–8.

5 A. L. Spek, *Acta Crystallogr D Biol Crystallogr*, 2009, **65**, 148–155.

6 M. J. Frisch, G. W. Trucks, H. B. Schlegel, G. E. Scuseria, M. a. Robb, J. R. Cheeseman, G. Scalmani, V. Barone, G. a. Petersson, H. Nakatsuji, X. Li, M. Caricato, a. V. Marenich, J. Bloino, B. G. Janesko, R. Gomperts, B. Mennucci, H. P. Hratchian, J. V. Ortiz, a. F. Izmaylov, J. L. Sonnenberg, Williams, F. Ding, F. Lipparini, F. Egidi, J. Goings, B. Peng, A. Petrone, T. Henderson, D. Ranasinghe, V. G. Zakrzewski, J. Gao, N. Rega, G. Zheng, W. Liang, M. Hada, M. Ehara, K. Toyota, R. Fukuda, J. Hasegawa, M. Ishida, T. Nakajima, Y. Honda, O. Kitao, H. Nakai, T. Vreven, K. Throssell, J. a. Montgomery Jr., J. E. Peralta, F. Ogliaro, M. J. Bearpark, J. J. Heyd, E. N. Brothers, K. N. Kudin, V. N. Staroverov, T. a. Keith, R. Kobayashi, J. Normand, K. Raghavachari, a. P. Rendell, J. C. Burant, S. S. Iyengar, J. Tomasi, M. Cossi, J. M. Millam, M. Klene, C. Adamo, R. Cammi, J. W. Ochterski, R. L. Martin, K. Morokuma, O. Farkas, J. B. Foresman and D. J. Fox, 2016, Gaussian 16, Revision B.09, Gaussian, Inc., Wallin.

7 A. D. Becke, *Phys Rev A (Coll Park)*, 1988, **38**, 3098–3100.

8 C. Lee, W. Yang and R. G. Parr, *Phys Rev B*, 1988, **37**, 785–789.

9 A. D. Becke, *J Chem Phys*, 1993, **98**, 5648–5652.

10 S. Grimme, S. Ehrlich and L. Goerigk, *J Comput Chem*, 2011, **32**, 1456–1465.

11 J.-D. Chai and M. Head-Gordon, *Physical Chemistry Chemical Physics*, 2008, **10**, 6615–6620.

12 *Schrödinger Release 2021-2: Maestro, Schrödinger, LLC, New York, NY, 2021.*

13 C. Lu, C. Wu, D. Ghoreishi, W. Chen, L. Wang, W. Damm, G. A. Ross, M. K. Dahlgren, E. Russell, C. D. Von Bargen, R. Abel, R. A. Friesner and E. D. Harder, *J Chem Theory Comput*, 2021, **17**, 4291–4300.

14 C. C. Lam and J. M. Goodman, *J Chem Inf Model*, 2023, **63**, 4364–4375.
